# From sequences to strategies: Early detection of new SARS-CoV-2 variants via genetic distance to reduce hospitalizations

**DOI:** 10.1371/journal.pcbi.1014707

**Published:** 2026-09-01

**Authors:** Marika D’Avanzo, Aung Pone Myint, Giacomo Cacciapaglia, Stefan Hohenegger, Francesco Conventi, Marta Nunes

**Affiliations:** 1 PhD National Programme in One Health approaches to infectious diseases and life science research, Department of Public Health, Experimental and Forensic Medicine, University of Pavia, Pavia, Italy; 2 INFN Sezione di Napoli, Complesso Universitario di Monte S. Angelo Edificio 6, Naples, Italy; 3 Center of Excellence in Respiratory Pathogens (CERP), Hospices Civils de Lyon (HCL) and Centre International de Recherche en Infectiologie (CIRI), Équipe Santé Publique, Épidémiologie et Écologie Évolutive des Maladies Infectieuses (PHE3ID), Inserm U1111, CNRS UMR5308, ENS de Lyon, Université Claude Bernard Lyon 1, Lyon, France; 4 Laboratoire de Physique Théorique et Hautes Energies (LPTHE), UMR, Sorbonne Université & CNRS, France; 5 Quantum Theory Center (ℏQTC) at IMADA & D-IAS, Southern Denmark Univ., Odense M, Denmark; 6 Université Claude Bernard Lyon 1, CNRS/IN2P3, IP2I UMR 5822, Villeurbanne, France; 7 Dipartimento di Ingegneria, Università degli studi di Napoli Parthenope, Centro Direzionale di Napoli, Naples, Italy; University of Western Ontario: Western University, CANADA

## Abstract

The COVID-19 pandemic highlighted the critical need for robust methods to monitor viral evolution and detect emerging variants of concern (VOCs). This study expanded an unsupervised clustering algorithm, based on Levenshtein distance, to track and predict variant predominance across six European countries from 2020 to January 2024. We also investigated the influence of genetic distances and containment strategies on hospitalization rates. Spike protein sequences were transformed into temporal chains. A deep neural network (DNN) was trained to classify emerging chains as likely dominant, while a CatBoost model assessed important variables, and simulations explored modifying vaccine genetic distance, containment measures, and vaccination coverage. Approximately 5,000 sequences per week enabled early chain detection within four weeks. The DNN achieved high classification performance for identifying future predominant chains within 3–4 weeks of detection. Genetic distance metrics between consecutive chains and between circulating and vaccine strains were among the most informative variables associated with hospitalization patterns. Model-based simulations suggested that scenarios involving improved vaccine matching or stronger containment measures were associated with lower predicted hospitalization burdens. Doubling vaccination coverage alone had minimal effect but showed additional reductions when combined with strict containment. Our findings from this integrated framework highlight the potential relevance of genetic distance metrics and public health interventions when assessing hospitalization risk associated with emerging variants.

## Author summary

In this study, we developed a new way to track how viruses like the one that causes COVID-19 change over time and predict which new versions might become widespread. During the pandemic, it became clear that we needed better tools to spot dangerous new variants early. By analyzing the genetic patterns of the virus’s spike proteins across six European countries from 2020 to early 2024, we created a computational model that can identify a rising variant within just three to four weeks of its first appearance. Beyond just tracking the virus, we investigated what actually drives hospitalizations. We found that the genetic distances between the current virus and previous versions and the distance with the vaccines correlated with how many people were hospitalized. Our simulations suggested that a significant impact on hospitalizations came from updated vaccines that closely matched the circulating virus and the public health measures. By providing a faster, more accurate way to monitor these genetic shifts, our framework offers a vital tool for health officials to make better-informed decisions and prepare for future outbreaks. From Sequences to Strategies: Early Detection of New SARS-CoV-2 Variants via Genetic Distance to Reduce Hospitalizations.

## Introduction

Severe acute respiratory syndrome coronavirus 2 (SARS-CoV-2) has evolved continuously producing variants with different levels of transmissibility, immune escape, and disease severity. Its spike protein is central to host cell entry and continuous accumulation of mutations in the spike protein have led to the emergence of phylogenetically distinct variants [1] which can evade immune responses from prior infections or vaccination [2]. Four of these variants, Alpha, Beta, Delta, and Omicron, have been classified as variants of concern (VOCs) by the World Health Organization (WHO), while others have been designated as variants of interest (VOIs) or variants under monitoring (VUMs) [3]. Since December 2021, Omicron and its sub-lineages have globally dominated the epidemiological dynamics. On May 5, 2023, the WHO declared the end of the COVID-19 public health emergency of international concern while acknowledging the ongoing risks posed by future SARS-CoV-2 evolution [4]. This highlights the importance of continuous viral genomic surveillance to promptly detect and assess new variants that could impact public health, particularly in the context of diverse immunity profiles resulting from varied exposure due to irregular vaccines coverage and prior infections.

To address this need, de Hoffer et al. [5] developed an unsupervised machine learning (ML) algorithm to define new variants by clustering the amino acid sequences of the spike protein of SARS-CoV-2 based on the Levenshtein distance, and a time-binned hierarchical clustering with Ward’s method. Clusters across consecutive time bins are then linked into temporal ‘chains’, representing the longitudinal evolution of genetically related viral variants over time, when they contain the same dominant spike sequence. These chains were empirically found to correspond to persistent and potentially epidemiologically significant variants. The approach effectively predicted the Alpha and Delta variants in the United Kingdom.

Levi et al. [6] analyzed data from 30 countries to identify variants associated with over 1,000 cases per million population within a three-month period using Jaccard distance. However, the method’s reliance on retrospective metrics, the maximum weekly case count observed over the full variant duration, limits its applicability for real-time prediction. Nicora et al. [7] used k-mer counts and a one-class Support Vector Machine (SVM) to flag anomalous sequences, though it suffered from a high false-positive rate. Rancati et al. [8] also employed spike k-mer representations with an autoencoder to predict lineages that would exceed 10% of total sequences, but its performance was inconsistent in countries with relatively lower sequencing volumes, such as France and Denmark. More recently, Feng et al. [9] developed a transformer-based model designed to forecast future lineage frequencies up to two months in advance, however, it relies on prior lineage designation and focuses exclusively on frequency forecasting. While these tools focus on detection and frequency prediction, recent studies have explored generative approaches aimed at producing plausible future viral sequences that may later become dominant. For example, the SARITA model [10] introduced a generative Large Language Model (LLM) designed to anticipate future mutations in the S1 subunit of the spike protein before they emerge in nature, successfully predicting hallmark mutations of variants like Delta and Omicron. Including such sequence-generation frameworks provides a more complete overview of the landscape of prospective genomic surveillance.

While COVID-19 vaccines have been shown to protect against infections and hospitalizations [11], the Omicron variant with extensive spike mutations has caused substantial hospitalizations [12] despite lower intrinsic virulence [13]. Recent research supporting this statement includes comparative analyses across European nations which highlight how the interplay between high-infectivity variants like Omicron and varying vaccination rates complicated pandemic management and epidemiological outcomes [14]. Current approaches for assessing vaccine effectiveness against novel variants (primarily in vitro neutralization assays [15,16]) have limitations in their timeliness and predictive capacity. Studies such as Cao et al. [17] have explored the relationship between genetic divergence and vaccine effectiveness, suggesting the genetic distance between circulating and vaccine strains as an indicator for immune escape potential. However, a direct, quantitative link between genetic divergence and real-world clinical outcomes has not been established.

In this study, we extend the unsupervised ML algorithm by de Hoffer et al. [5] to analyze spike protein sequences from six European countries. We assess the robustness of the algorithm across geographically and temporally diverse datasets, identify the optimal sequence volume required for early variant detection and evaluate predictive parameters for variant predominance. Furthermore, we incorporate a deep learning classifier to improve early predictions of variant dominance based on early prevalence trends. Additionally, we investigate impact of genetic distance between variants and distances with vaccine strains on observed hospitalizations by leveraging comprehensive Danish health data, together with different containment strategies. Our framework aligns with recent findings that emphasize the need for integrated models to understand the complex interdependencies between viral evolution, public health policies, and vaccination strategies [14]. Rather than introducing an entirely new clustering methodology, the novelty of this work lies in integrating cross-country chain surveillance, early-stage predominance prediction, and hospitalization-associated genetic distance analysis into a unified predictive framework. By integrating the identification of emerging variants through genetic surveillance with a novel analysis of the direct link between spike protein evolution and hospitalization, this study offers a valuable framework for predicting and responding to future epidemics caused by novel viruses.

## Results

### Cross-country variant surveillance

A total of 2862331 sequences for the six countries were analyzed, resulting into 109 chains for Germany, 142 chains for Italy, 95 chains for Sweden, 82 chains for Denmark, 89 chains for France and 98 chains for Spain. A total of 10 chains from Germany, 11 chains from Italy, 8 chains from Sweden, 8 chains from Denmark, 7 chains from France and 12 chains from Spain were selected and successfully matched with the circulating variants. Temporary interruptions in chain detection, typically associated with short-term reductions in sequencing availability, were resolved using predefined merging criteria based on dominant-sequence identity or Ward-distance continuity (Methods).

**Fig 1:**
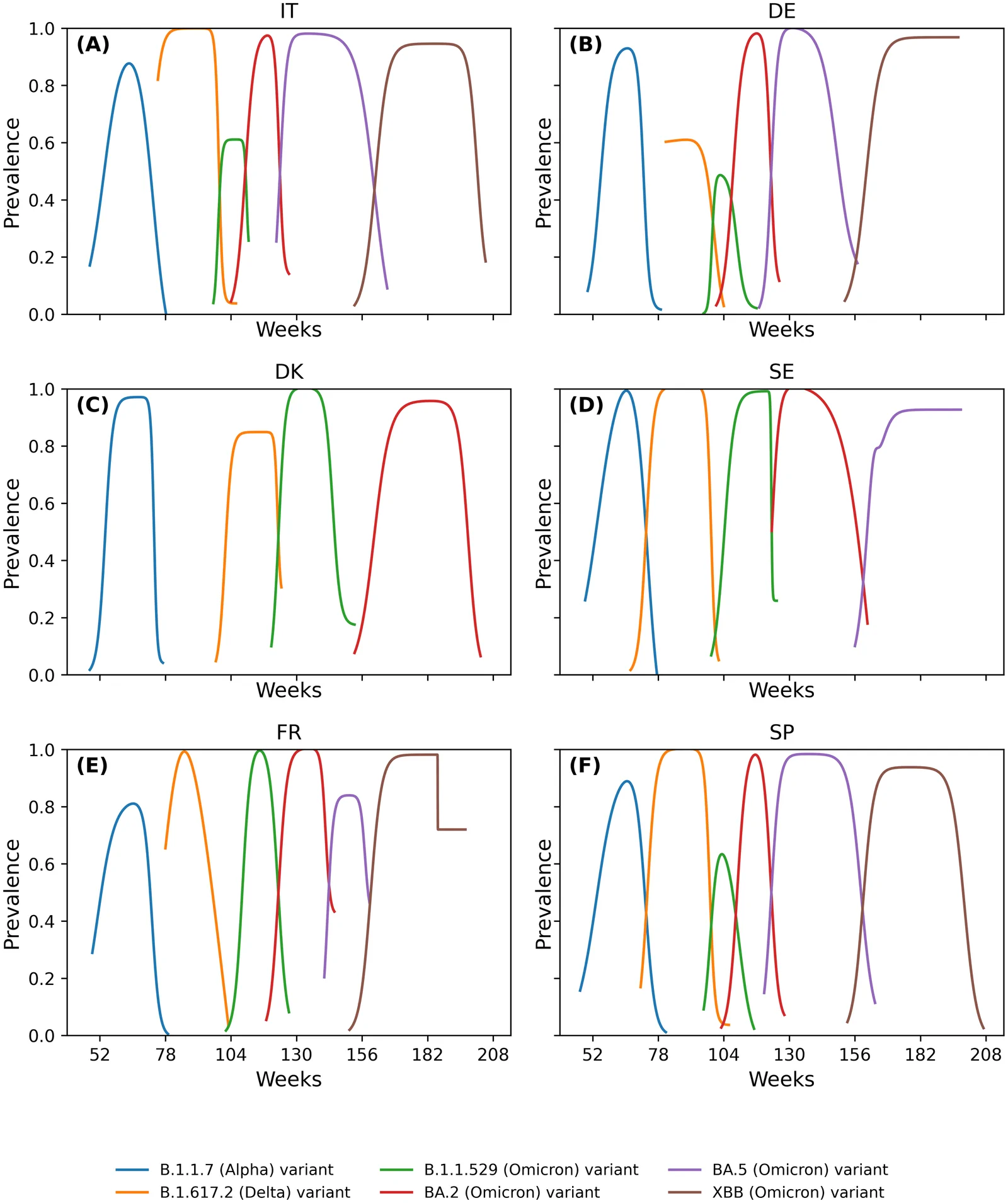
Fitted chains for Italy (a), Germany (b), Denmark (c), Sweden (d), France (e), and Spain (f) from 2020 to 2023. For each country, chains representing the evolution of a variant were fitted using a combination of two sigmoid functions to capture the increase and decrease phase of variant prevalence. On the x-axis, time in weeks is plotted, while on the y-axis the percentage of prevalence (from 0 to 1) of the variant of the chain with respect to all the sequences is considered. Chains are numbered by the algorithm and numbers are assigned according to the number of the first cluster of that chain [5].

Comparison of the fit results for the prevalence time evolution for the six considered European countries is reported in Fig 1. The direct comparison of the six main variants across various countries highlighted that the time evolution dynamics of each variant was largely independent of the geographic location (Fig A in S1 text).

The growth rate parameters obtained from the fit at weeks 3 and 4 ($a_{\text{3}}$ and $a_{\text{4}}$) differed between dominant and transient chains, and may serve as early indicators of a chain’s long-term behavior. In dominant chains such as France chain 221, the parameters (a) the growth rate, (b) the inflection point (point of change in the trend), and (L) the upper asymptote (maximum expected prevalence) showed stability after a few weeks in comparison with non-dominant chains like Denmark chain 169 with ongoing fluctuations (Fig B in S1text).

**Fig 2:**
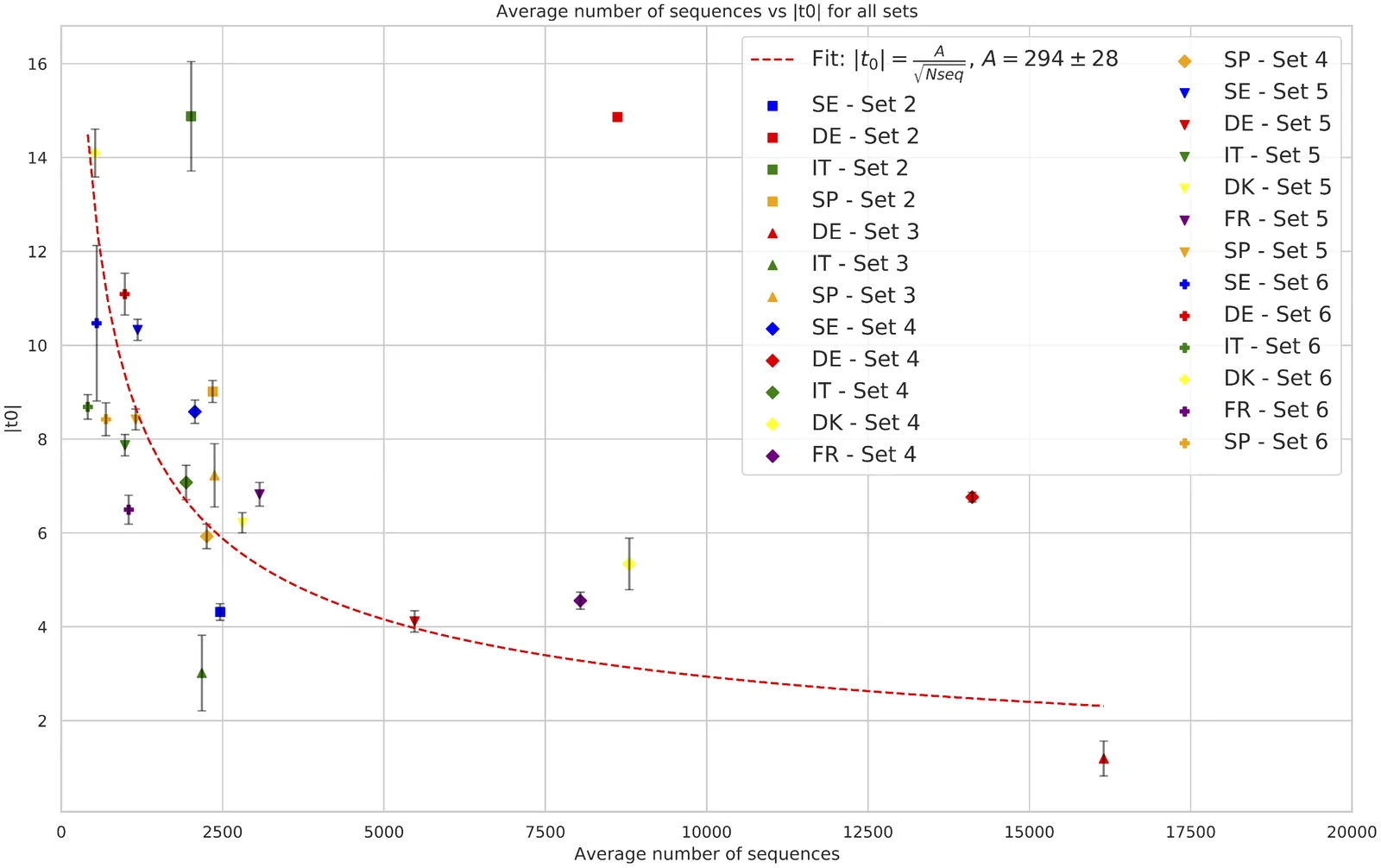
Calibration curve. Each point corresponds to a variant detected in a specific country, with marker shape indicating the set and color indicating the country. Error bars represent uncertainty on |𝑡0| from the fitting procedure. A red dashed curve shows the best fit according to an inverse power law model, |t0| = A/√x, where x is the average weekly number of sequences. The fitted parameter is A = 294 ± 28.

The fitted curve provided the magnitude of sequencing effort required to minimize $t_{\text{0}}$ (time taken to isolate the chain) and enhance early variant detection (Fig 2), and approximately 5000 sequences per week were needed for the benchmark, $t_{\text{0}}=\text{4}$.

### Deep learning-based early detection of predominant variant

The initial dataset for the deep neural network (DNN) model was composed of 37 predominant chains and 78 transient chains. The models trained on the final dataset including simulated chains showed a very low False Positive Rate (FPR) value even at a very high value (99%) of high True Positive Rate (TPR) for all the three scenarios using the full set of parameters (Table A in S1 Text). A simplified network obtained adding only growth-related parameters ($a_{\text{3}}$, $b_{\text{3}}$, $L_{\text{3}}$) also significantly improve the prevalence-only model accuracy.

Receiver Operating Characteristic (ROC) curves confirmed strong overall classification performance, with growth-related parameters offering clear advantages in sensitivity, especially in the more restrictive Case 3 dataset (Fig C in S1 text). However, relying solely on prevalence values implies a performance drop.

**Fig 3:**
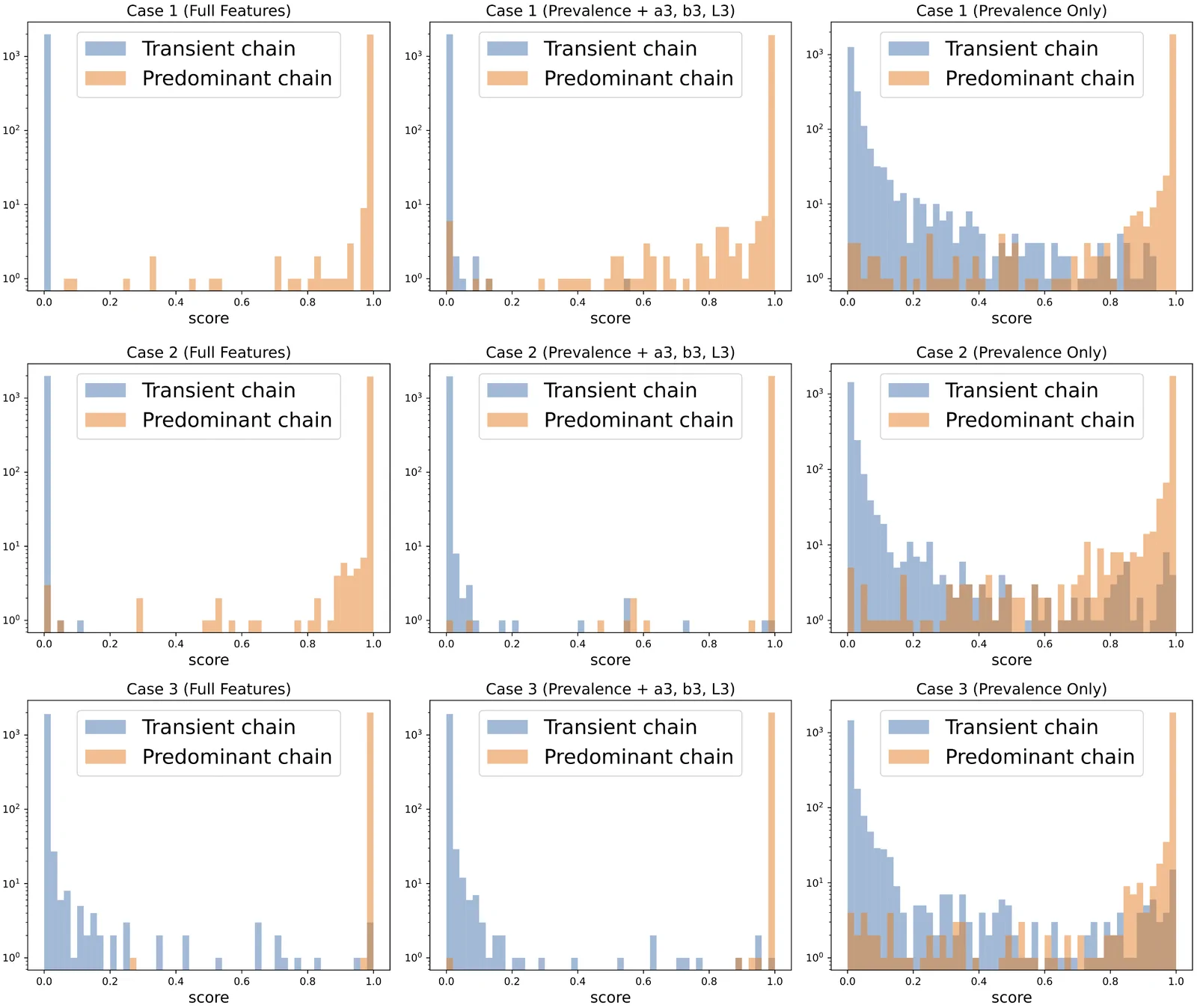
DNN score distribution comparison across dataset configurations and feature sets. The classifier score represents the predicted probability that a chain will become predominant. Predominant chains are expected to accumulate near scores close to 1, whereas transient chains accumulate near 0. The signal-to-noise ratio (i.e., Predominant chains to Transient chains ratio) quantifies the separation between true positive and false positive predictions for different classifier scores. Higher separation between the two distributions indicates better discriminative performance of the classifier. Including growth-related parameters consistently improves the signal-to-noise ratio and consistently improves class separation and reduces overlap between transient and predominant chains, particularly in the more challenging low-prevalence Case 3 dataset, where prevalence-only models show reduced predictive power.

The DNN score distribution further highlighted the effective discriminative power of the deep learning model across all datasets except in the model with prevalence data only (Fig 3). A summary of the classification performance of the deep learning model across all scenarios, considering both different feature subsets and increasing levels of data restriction, can be found in the Fig D in S1 text.

### Validation on real-world dataset

To assess whether the deep neural network learned meaningful patterns beyond the synthetic chains used for data augmentation, that transfer to real epidemiological observations, we evaluated the final model on real chains extracted from the six-country surveillance dataset. Although these chains contributed to the estimation of the distributions used for synthetic data generation, they were not presented directly to the classifier during training. The model was trained using the same procedure described above and subsequently evaluated on a dataset of real-world chains. After excluding three chains due to incomplete information in the sequencing data, the validation dataset consisted of 111 real chains (78 transient and 33 predominant). Since the full-feature model consistently achieved the highest performance across all evaluated feature configurations, only the results of the final selected model are presented here.

The classifier maintained a high level of performance on this real-world dataset, achieving an overall accuracy of 97.3% and a ROC-AUC of 0.980. Only three chains were misclassified: two transient chains were incorrectly predicted as predominant and one predominant chain was incorrectly classified as transient (See Fig E in S1 Text).

Class-specific performance remained high for both categories. Predominant chains achieved a precision of 0.94, recall of 0.97, and F1-score of 0.96, while transient chains achieved a precision of 0.99, recall of 0.97, and F1-score of 0.98.

The distribution of prediction scores showed a clear separation between transient and predominant chains, with most transient chains assigned probabilities close to 0 and predominant chains assigned probabilities close to 1. This result suggests that the model captures discriminative patterns that generalize to real epidemiological observations and are not solely driven by the synthetic augmentation procedure.

Additional robustness checks were performed by re-fitting parameter distributions after excluding individual countries from the dataset, which confirmed that distributional stability and model fitting were preserved across all cases (Figs F, G, H and I in S1 Text).

### Analysis of hospitalizations by variants in Denmark

**Fig 4:**
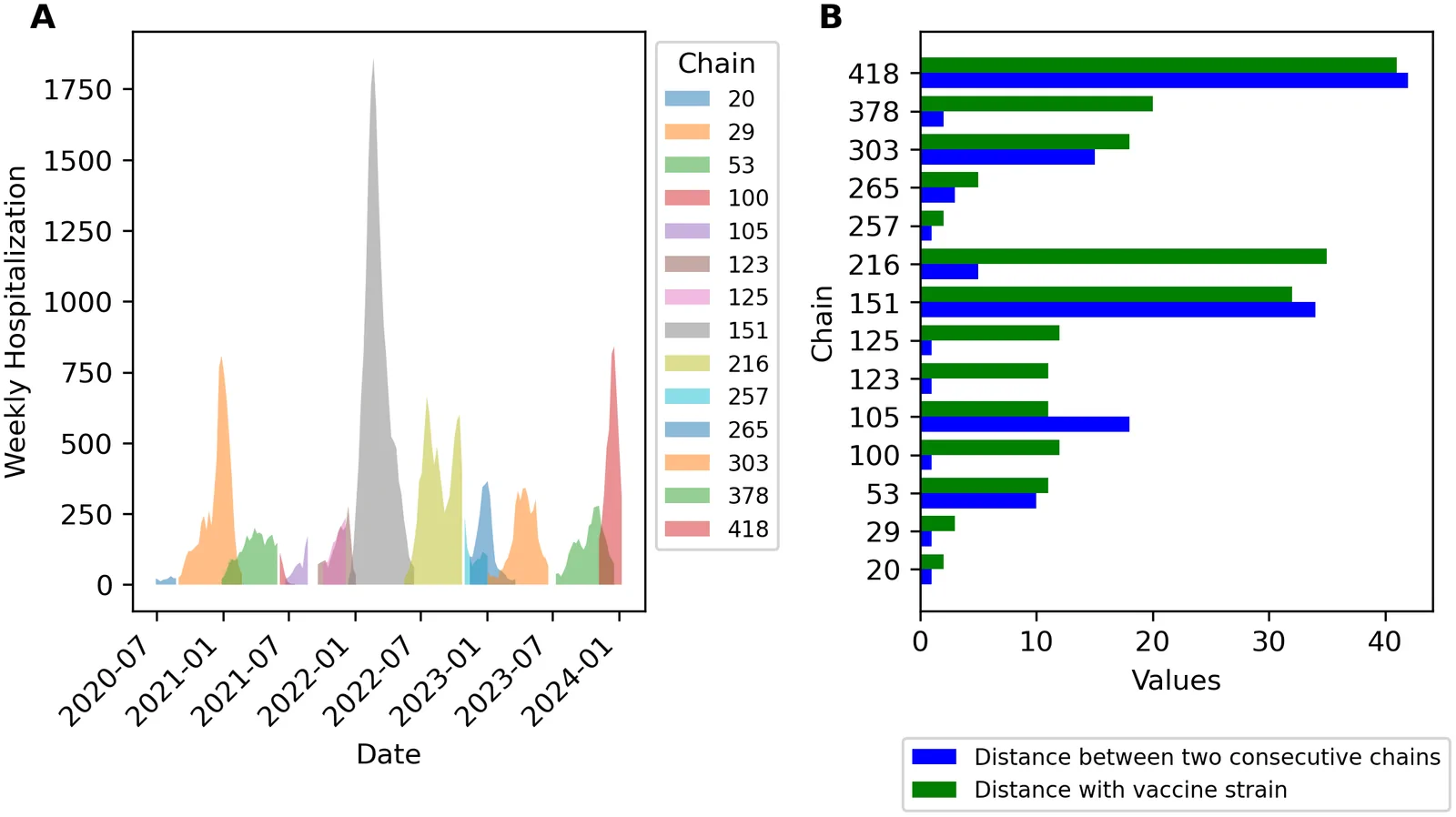
Weekly hospitalizations and Levenshtein distances between consecutive chains and their respective vaccine strain distances for each chain, observed in Denmark from July 2020 to January 2024. Variant classifications: Chain 53 – Alpha, 105 – Delta, 151 – Omicron BA.2, 216 - BA.5, 303 – XBB.1.5, 418 – JN.1.

14 chains from Denmark were included, and 56,345 (80% of total) hospitalizations were observed by these chains. Weekly hospitalizations by each chain were shown in Fig 4. High distances were observed during the emergence of major variants such as Alpha (chain 53), Delta (105), and the Omicron sublineages BA.2 (151), XBB.1.5 (303), and JN.1 (418) (Fig 4). The reinfection rate, Containment and Health Index (CHI), vaccination coverage (VCR) at each week, and distribution of interaction of the lagged CHI with the detection week (CH_week) can be found in Figs J and K in S1 Text.

The CatBoost model demonstrated high predictive accuracy, achieving an overall R² score of 0.98 and 0.85 on the unseen test dataset. The mean absolute error (MAE) was 0.10 for the training set and 0.31 for the test set. Despite the higher error on the test data, the magnitude remained small when compared with the outcome with a minimum of 0.52 and an average of 4.90. Residual diagnostics showed no evidence of heteroscedasticity or non-linearity (Fig L in S1 Text).

**Fig 5:**
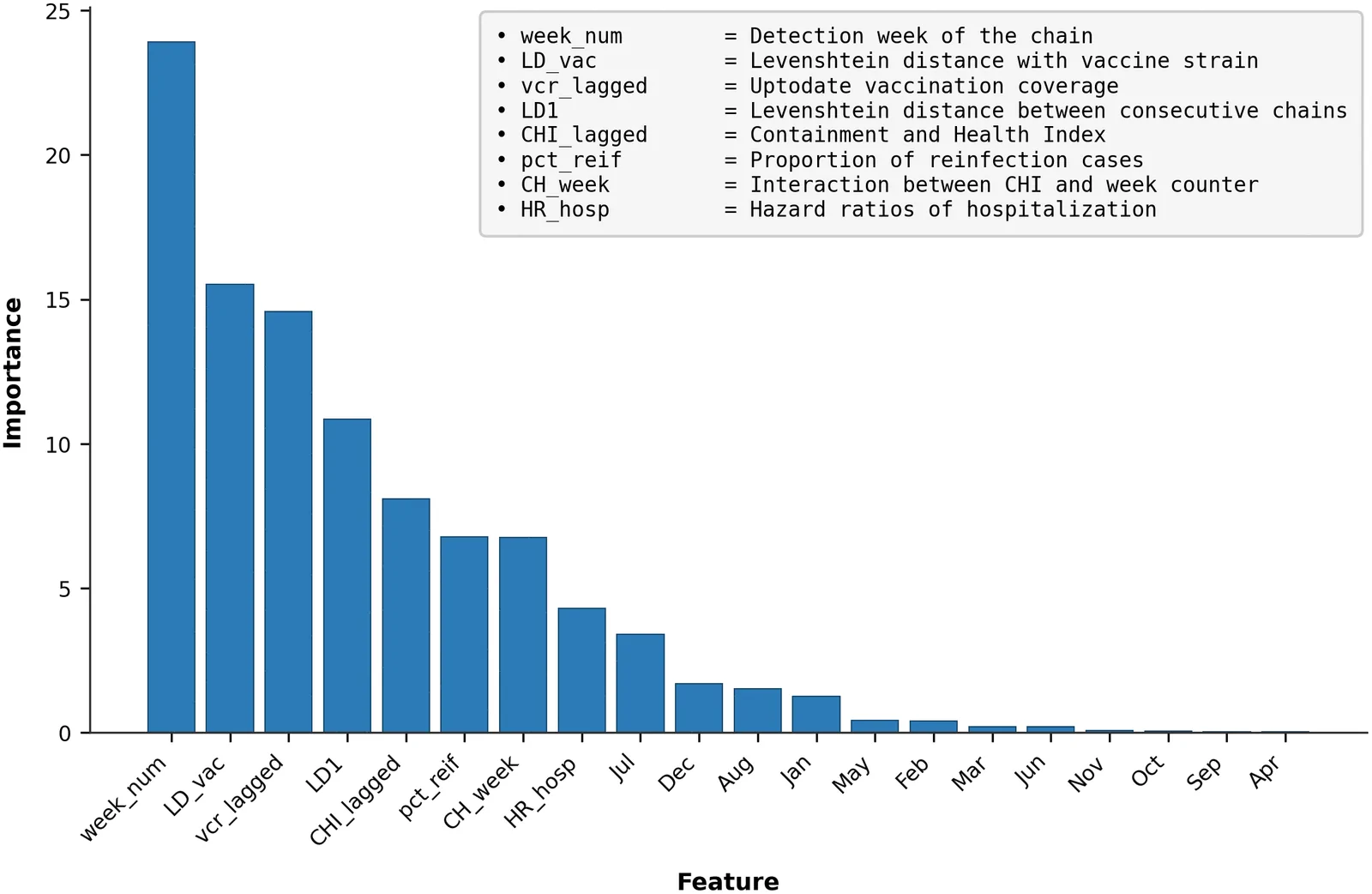
Feature importance of the CatBoost model for prediction of weekly hospitalizations by chains in Denmark (Jul 2020 - Jan 2024).

The detection week of each chain was the most impactful variable, contributing 23.9% to the model’s explanatory power, followed by the Levenshtein distances between dominating sequence and the vaccine strain (LD_vac) with 15.5%, two weeks legged up-to-date VCR (vcr_lagged) with 14.6%, and Levenshtein distances between dominating sequences of the consecutive chains (LD1) with 10.8% (Fig 5). The SHAP (SHapley Additive exPlanations) plot (Fig M in S1 Text) indicated that the week counter showed the highest impact on the outcome with top position, and exhibiting bell-shaped association in the partial dependence plot (Fig N in S1 Text). High LD1 and LD_vac values indicated a positive relationship with hospitalizations and high impact on the outcome, while negative association was observed for two weeks lagged CHI (CHI_lagged). Finally, hospitalizations also showed seasonal trends, increasing during December and January and decreasing during July and August (Fig O in S1 Text).

While simulating with better-matched vaccines, significant reductions in hospitalizations (26–55%) were observed for chains 151, 216, and 418 while nonsignificant reductions observed for chain 303 and no change for chain 378. Temporary or sustained CHI had minimal impact on early waves chain until 123 observed before 2022, since CHI levels were already high during these periods (average CHI > 50). Doubling VCR alone produced no statistically significant changes in hospitalization trends across any of the analyzed chains. Additional reductions in hospitalizations were observed in high CHI simulations when combined with doubling VCR.

**Fig 6:**
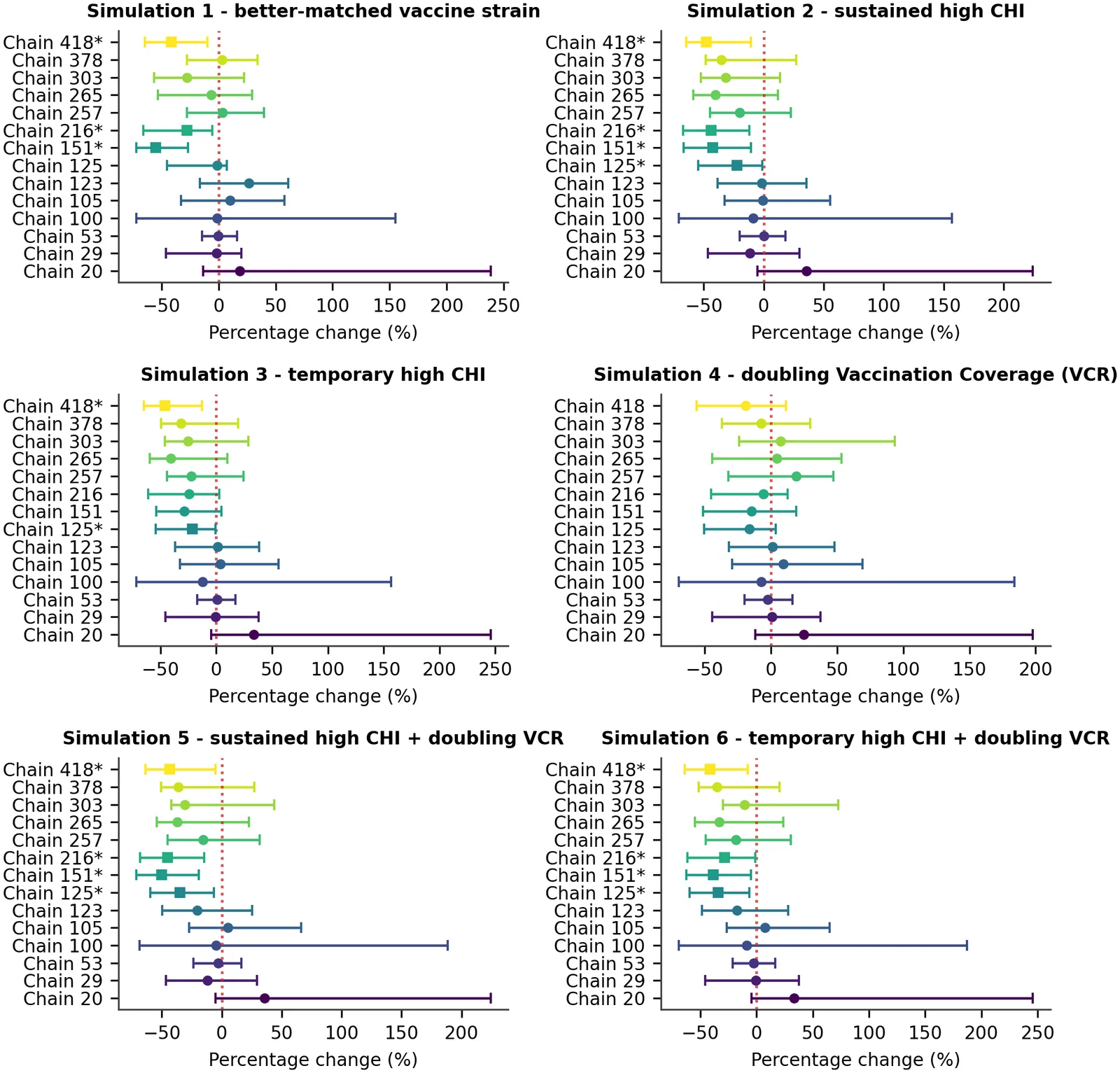
Changes in the proportion of total hospitalizations per chain in Denmark, across different scenarios, with respective 95% confidence intervals (CIs). Significant changes were shown with asterisk (*).

Details of the simulated impact on the reduction in the percentages from total hospitalizations are presented in Fig 6, and detail simulations of weekly hospitalizations and cumulative hospitalizations can be found in Figs P,Q, and R in S1 Text.

Sensitivity analyses revealed that including the genetic distance improved the model performance compared with the models without those variables. The models with different lagged proportions of variants also indicated that the top four important features (the weekly counter, vaccination coverage, and the genetic distances) were consistently showing as the most important features. Details of the results can be found in the Figs S and T in S1 Text.

## Discussion

This study highlights several critical insights into the optimization of SARS-CoV-2 surveillance strategies and enabling proactive variant detection and response. It was found that approximately 5000 sequences per week for detection within 4 weeks target enables timely detection and facilitates early intervention measures, and provides a universal guideline for sequencing requirements. Currently, reporting of SARS-CoV-2 sequences in GISAID has been diminishing sharply in all countries, and reached lowest point in July 2025 with 7,802 sequences [18].

Unlike traditional surveillance, our DNN model rapidly identified dominant variants using only the first few weeks of prevalence data. It improved this assessment by integrating sigmoid growth parameters and their derivatives. Our findings with low initial-prevalence chains only even showed similar (or even better) classification performance, supporting the idea that early-stage variant detection is feasible even when initial chain prevalence is quite low, which is critical for timely public health responses.

These insights highlight a key advantage of deep learning over rule-based classification or threshold-based methods. Instead of relying on arbitrary cutoffs for identifying concerning variants, our model learned from observed epidemiological dynamics, allowing for data-driven decision-making. Compared to previous studies our model demonstrates improved performance [7,8], usability in real-time monitoring [6], and the adaptability and reliability in scenarios with limited sequencing data [8].

Our fundings in Denmark support the hypothesis that vaccine matching and genetic monitoring may provide useful information for anticipating hospitalization trends associated with emerging variants. The distances between consecutive variants and distance from the vaccine strain were among the most significant features correlated with the weekly hospitalization rates, which was supported by reduced vaccine effectiveness for new variants [19,20]. While Denmark possesses a unique demographic and healthcare profile including high coverage of disease surveillance, it serves as a powerful proof-of-concept for how these strategies can be deployed globally.

Across all evaluated models, temporal progression (represented by the week_num feature) emerged as the strongest predictor of weekly hospitalizations. This robust temporal dependency is directly tied to our study design, which focused specifically on predominant variants. Because predominant variants characteristically follow a predictable epidemiological trajectory, typically manifesting as a classic bell curve, time naturally becomes the primary driver of hospitalization volume. Consequently, the temporal feature captures the underlying life cycle of the variant wave, rendering it the most critical variable in our predictive models.

Our simulations indicated a clear correlation between genetic distance and clinical outcomes at a population level, except in one case, chain 378, which was first reported just before the introduction of the matched XBB.1.5 vaccine. Building upon this, our finding highlights that tracking genetic distance may provide complementary information for situational awareness and hospital resource planning., even without complete epidemiological data in the emergence of unknown variants. While real-world delays in vaccine production and approval make the immediate deployment of a closely matched strain a purely theoretical scenario, these findings validate the essential role of vaccine matching and endorse the continued development of variant-updated COVID-19 vaccines.

Simulated scenarios incorporating stronger non-pharmaceutical interventions were associated with lower predicted hospitalization burdens., and combining with doubling VCR showed additive benefits, which suggests that a multifaceted approach could yield even greater benefits in the absence of matched vaccines. The observed reductions, particularly in chains with lower initial containment levels or delayed vaccine strain matching, emphasize the importance of timely public health measures in the emergence of genetically distinct variants like chains 151 (BA.2), 216 (BA.5), and 418 (JN.1).

These insights provide a compelling case for sustained investment in genomic surveillance, vaccine development, and integrated public health responses to safeguard against the evolving threat of SARS-CoV-2 and other emerging pathogens.

Several limitations should be taken into account in our study. First, the performance of both the clustering algorithm and the deep learning model was inherently dependent on the availability of genomic data, and may introduce biases due to sequencing delays, particularly during the early emergence of new variants. In addition, sequencing intensity varied substantially across countries and across different phases of the pandemic, potentially affecting early chain detection and prevalence estimation. Differences in national surveillance strategies, reporting practices, and sampling representativeness may therefore influence the generalizability of the framework across epidemiological settings. Second, the predictive power of the DNN relied on features extracted during the first few weeks after a variant’s initial detection from the clustering algorithm, and variants with delayed or irregular dynamics may challenge the model’s classification ability. Thirdly, the data augmentation strategy might not fully capture the diversity of real-world evolutionary behaviors, particularly for variants exhibiting novel or outlier dynamics. In addition, the number of available real-world predominant chains remains relatively limited compared to the size typically used for deep neural network training, which may affect the stability and prospective generalizability of the classifier despite the strong performance observed on the real-chain validation dataset. Although the real-chain validation demonstrated strong classification performance, the synthetic augmentation procedure may still favor dynamics that resemble the assumptions used during data generation. Therefore, prospective validation on future emerging variants remains necessary. Fourthly, its ability to predict the trajectory of newly emerging variants, especially different viruses, in real-time remains to be systematically validated. Although the validation on real-world chains supports the ability of the model to generalize beyond synthetic augmentation, future work should include fully prospective evaluations using temporally separated datasets or leave-one-country-out validation strategies to further assess robustness across heterogeneous epidemiological settings.

Future research should explore integrating real-time data streams, improving robustness against sequencing gaps, and evaluating transferability to other viruses. Fifth, our conclusions regarding hospitalization may lack external validity, given that they are derived from the distinct regional dynamics and unique healthcare system of Denmark and rely on theoretical modeling implementations. Another limitation is that the proportion of variants among hospitalized patients might be different from that in the general infected population. This discrepancy may arise from differential testing practices, demographic differences, vaccination status, healthcare-seeking behavior, or preferential sequencing of specific patient groups, potentially introducing systematic biases in the hospitalization attribution procedure. However, focusing on major variants in the model may partially address this issue, as these variants are more likely to be associated with higher hospitalization rates. The consistent identification of the same top important features across various models also supports that the initial assumptions on variant proportions had a minimal impact on the primary findings. Additionally, we were unable to incorporate detailed population structures of vaccine recipients or hospitalized patients due to data availability constraints. Furthermore, the long-term impact of vaccinations from previous seasons on hospitalization rates was not included in our analysis. While this omission may not significantly alter outcomes, it remains a factor worth exploring in future studies to ensure a more comprehensive understanding. Finally, modifying key variables within the CatBoost model introduced wide CIs resulting many scenarios’ reduction insignificant. Future research should aim to precisely quantify the relationship between the magnitude of variable modification and the resulting predictive accuracy, thereby refining the ability to generate nuanced and robust hospital burden forecasts. This would not only improve the interpretation of scenario-based predictions but also support the development of more resilient and informative forecasting methodologies. Finally, the hospitalization analyses and scenario simulations should be interpreted as exploratory and predictive rather than causal. The CatBoost model captures statistical associations present in the data and does not provide a formal causal framework for estimating the effects of interventions such as vaccination strategies or containment policies.

## Conclusion

The integration of optimized sequencing rates, early-stage classification with deep learning, and genetic-based hospitalization risk assessment offers a powerful, data-driven framework for SARS-CoV-2 surveillance. We show that adequate sequencing volume (impacting on the time needed to detect chains) together with classification models based on growth dynamics and incorporating genetic distance metrics into surveillance frameworks may improve the characterization of emerging variants and support public health decision-making. We demonstrate that early variant classification is feasible using only a few weeks of prevalence data and highlighting the utility of sigmoid-based features in improving classification accuracy even with low initial-prevalence chains. We illustrate a potential correlation between genetic distance and hospitalization risk in Denmark, supporting its potential use as a complementary surveillance signal for situational awareness in public health decision-making and we highlight the need for integrated deep learning, genetic monitoring, and vaccination strategies for pandemic preparedness.

These findings contribute to a growing framework for real-time, adaptive surveillance systems that can rapidly respond to emerging epidemiological threats. Future work will focus on enhancing the robustness of the DNN model, exploring alternative architectures (e.g., transformer-based models), and refining transient chain modeling techniques to further improve predictive accuracy.

## Materials and Methods

### Ethical statement

This study exclusively utilized publicly available, anonymized data, and therefore, no direct human subjects research was conducted. As such, formal ethical approval from an Institutional Review Board (IRB) or equivalent ethics committee was not required. All data sources were accessed and analyzed in accordance with their respective terms of use and public accessibility guidelines. Care was taken to ensure that the analysis of this publicly available data did not inadvertently reveal personal or sensitive information of individuals, maintaining the anonymity and privacy inherent in the original datasets.

### Data sources

We analyzed SARS-CoV-2 Spike protein sequences from Germany, Italy, Sweden, Denmark, France, and Spain sourced from the GISAID database [18]. The Oxford Covid-19 Government Response Tracker provided data on CHI through the end of 2022 [21]. Weekly hospitalization numbers ($H_{actual,t}$), reinfection percentages (pct_reif) and weekly VCR (${Primary}_{t},\;{Booster}_{t}$) in Denmark were obtained from the Denmark Statens Serum Institut (SSI) [22]and complemented by the European Centre for Disease Prevention and Control (ECDC) [23]. Variant-specific hospitalization risks (HR_hosp) were obtained from a retrospective study in Washington State covering December 2020 to January 2022 [24].

### Cross-country variant surveillance

Our analysis began with the algorithm developed by A. de Hoffer et al. [5], to identify viral chains with the data from spike protein sequences collected between January 23, 2020 and January 15, 2024. In this study, a “chain” refers to a temporally connected series of clusters representing the persistence and evolution of genetically related spike protein sequences across consecutive weeks. Chains that were observed at least four weeks and had a peak prevalence exceeding 20% were selected for analysis. To account for temporary interruptions in detection caused by fluctuations in sequencing coverage or sampling, chains separated by a gap of one to three weeks were evaluated for possible merging. Two interrupted chains were merged when at least one of the following criteria was satisfied:

(i) the dominant sequence of the downstream chain was identical to the dominant sequence of the upstream chain, corresponding to a strong link according to the chain-construction procedure described by de Hoffer et al. [5];
(ii) the dominant sequences were not identical but remained within the Ward-distance threshold used for cluster linking (distance ≤ 100), indicating genetic continuity between the two chains.

These criteria are consistent with the original chain-construction framework described by de Hoffer et al. [5] and were applied uniformly across all countries.

These chains were associated with the respective variants using spike protein sequences available from NCBI (National Center for Biotechnology Information, National Library of Medicine) and the CoVsurver app on GISAID.

We then fitted the frequencies of selected chains (P_c_) using a mathematical function of the time x to model their growth, with parameters L, *b, and a*, and decline, with L_2_, *b*_*2*_*, and a*_*2*_.


$$\begin{array}{c} P_{c}(x,a,b,L,a_{\text{2}},b_{\text{2}},L_{\text{2}})=L\left(\frac{\text{1}}{\text{1}+e^{\frac{-(x-b)}{a}}}\right)-L_{\text{2}}\left(\frac{\text{1}}{\text{1}+e^{\frac{-(x-b_{\text{2}})}{a_{\text{2}}}}}\right) \end{array}$$

Key parameters derived from this fitting included a, b, and L. When fitting only a limited number of weeks *k*, we labelled the parameters with a corresponding subscript (i.e., $a_{k}$)

All predictive features used for classification were computed exclusively from observations available within the first three or four weeks after chain detection. Information from later stages of chain evolution was not used during feature extraction.

We also derived a parameter $t_{\text{0}}$ that measures the time taken to isolate the chain ($t_{\text{0}}=b-\text{6}.\text{9}\text{1}\times a)$, by plotting the average number of sequences available per week against the absolute value of the first detection time, |𝑡0|, for variant chains across different country sets. A schematic representation of the main parameters used in this work has been reported in Fig U in S1 Text).

### Deep learning-based early detection of predominant variant

The dataset was constructed by identifying viral lineages that were observed for at least four consecutive weeks of sequence detection and could be used to derive the parameters needed for the model. Real-world chains from six countries were labeled as the predominant chains if they eventually reached ≥ 50% prevalence, and the rest as the “transient chains”.

Since real-world data for early-stage variant used to derive the parameters is limited, we applied a data augmentation strategy to generate synthetic chains. Synthetic chains were used exclusively for data augmentation during model training. Real chains extracted from the six-country surveillance dataset were instead reserved for downstream evaluation analyses aimed at assessing generalization to observed epidemiological dynamics.

For predominant chains, we fitted the key parameters with different distributions from real chains (Fig F in S1 Text), and generated new chains by sampling from these fitted distributions, adding a small noise using an exponential error model. The prevalence at week 1 was computed and the growth curve was recalculated iteratively. For transient chains, we classified them into two transient chain groups (Fig V in S1 Text): above-threshold (showing some growth tendency) and below-threshold (typically vanishing early). Weekly prevalence differences, expressed as the percentage of chains ($W_{i+\text{1}}-W_{i}$), were computed for both groups and modeled using Gaussian distributions. Chains were generated using an iterative procedure sampling weekly difference, ensuring non-negativity and mimicking early extinction events when the values dropped to zero.

We next applied a DNN model consisting of an input layer with 12 epidemiological features: prevalence at week 1,2, and 3, early-stage fit parameters ($a_{\text{3}},b_{\text{3}},L_{\text{3}},a_{\text{4}},b_{\text{4}},L_{\text{4}}$), and their first-order derivatives $(a_{\text{4}}^{\prime },b_{\text{4}}^{\prime },L_{\text{4}}^{\prime }$). It was followed by two hidden layers with 64 and 32 neurons respectively, both Rectified Linear Unit (ReLU) activation. The output layer utilized a sigmoid function for binary classification. Model training was carried out using the Adam optimization algorithm and binary cross-entropy as the loss function. The model was trained for 50 epochs using a batch size of 128. The training dataset was randomly split into 80% training and 20% validation. The real-world validation presented separately in the Results section was performed independently from this internal train-validation split and focused exclusively on observed epidemiological chains.

To evaluate the impact of the initial prevalence of the chains on the classification performance, we generated three different datasets: case 1 - all chains, case 2 – only predominant chains with 15% of prevalence at week 1, and case 3 – only predominant chains with 2% prevalence at week 1. Each dataset was generated to get 10,000 predominant chains and 10,000 transient chains chosen to allow a reasonable training of the models. DNN models were constructed for each dataset using different feature scenarios like using all twelve input features, prevalence values + $a_{\text{3}},b_{\text{3}},L_{\text{3}}$, or prevalence values only.

Model performance was evaluated using the ROC curve, the FPR values at high TPR thresholds, the confusion matrices, and the signal-to-noise score distribution patterns.

### Validation on real chain

To assess whether the classifier trained on synthetic examples generalized to observed epidemiological dynamics, we additionally evaluated the final model on real chains extracted from the six-country surveillance dataset. Chains for which the complete set of week-3 features could not be computed were excluded from the analysis. Specifically, three chains were removed because missing observations prevented reliable estimation of the growth-related parameters. The remaining real chains were classified using the trained model and performance was assessed using accuracy, ROC-AUC, confusion matrices, precision, recall, F1-score, and prediction score distributions.

### Analysis of hospitalizations by variants in Denmark

To investigate the relationship between SARS-CoV-2 genetic variation and hospitalization rates, we focused on Denmark due to the availability of comprehensive public data. We selected the chains with >3 week prevalence and ≥50% of prevalence at least at one time point to reduce noise from transient and low-prevalent chains. This approach ensures our analysis isolates the dominant variants that pose the most substantial risk of driving major, systemic hospitalization surges. Chains with the same dominating sequences were combined, even when gaps in their detection weeks led the model to classify them as separate. We tracked the progression of the variant chains using a weekly counter (week_num = t). We used the Levenshtein distances which calculates the exact number of single genetic mutations, including insertions, deletions, or substitutions, required to transform one viral sequence into another, to calculate the distances between dominating sequences of the consecutive chains (LD1). We also computed the distance between dominant sequence and the spike sequence used in the corresponding vaccine being employed at the time (LD_vac). We used the Wuhan strain (EPI_ISL_402124) sequence for the 2020–2021–22 season, the BA.5 strain (EPI_ISL_14026118) for 2022–2023 season as both BA.1 and BA.4/5 bivalent vaccines were introduced in that season [25,26], and the XBB.1.5 strain (EPI_ISL_16134259) for the 2023/24 season [27]. These distances represent the degree of mutational divergence between currently circulating variants and previous or vaccine-targeted strains. To estimate hospitalizations attributable to each variant chain, we assumed that the proportion of sequences belonging to a given chain in any week ($P_{seq,t}$) reflected its proportion of hospitalizations, which were then log-transformed ($log\left(\frac{P_{seq,t}\;\times \;H_{actual,t}}{\text{1}\text{0}\text{0}}\right)$). Other variables included in the analysis were 2-week lagged VCR if primary or booster doses were reported (VCR_lagged, ${VCR}_{t-\text{2}}=P\left({Primary}_{t-\text{2}}\;\cup \;{Booster}_{t-\text{2}}\right)$), 2-week lagged CHI which were projected to be 21.43 beyond 2022 as this magnitude had been sustained since April 2022 (CHI_lagged ${CHI}_{t-\text{2}}$), interaction of the lagged CHI with the detection week (CH_week = ${CHI}_{t-\text{2}}\;\times t$), and hospitalization risk due to variants in which sub-lineages of Omicron were considered as similar [28–31].

We used a CatBoost regression model to predict weekly hospitalizations per chain. The dataset was randomly split into 80% for training and 20% for testing. We fine-tuned key hyperparameters (tree depth, learning rate, L2 regularization, and the number of boosting iterations) to improve model performance and and evaluated it using 5-fold cross-validation to assess its robustness. Finally, the model’s predictive performance was assessed by comparing predicted hospitalizations to observed values, examining residual plots, and calculating R² and MAE. Feature importance scores, SHAP plot, and partial dependence plots were generated to understand each variable’s contribution.

We conducted sensitivity analyses to evaluate how different parameters and variant proportion assumptions impacted our results. First, we compared the performance (R2, MAE, MAPE, and RMSE) of three models: a time-only model using week_num and months (M1); the full model excluding the genetic distances (LD1 and LD_vac (M2); incorporating M1 model with genetic distances (M3); and the main model. Second, to test the sensitivity of our variant proportion assumptions, we compared the top feature importances of the main model with two models using one-week and two-week lags for weekly hospitalizations by variant chains ($log\left(\frac{P_{seq,t-\text{1}}\;\times \;H_{actual,t-\text{1}}}{\text{1}\text{0}\text{0}}\right)$ and ($log\left(\frac{P_{seq,t-\text{2}}\;\times \;H_{actual,t-\text{2}}}{\text{1}\text{0}\text{0}}\right)$).

Finally, we used the trained model to explore the impact of different scenarios: employing better-matched vaccine (distance at 5 for those with ≥15), implementing sustained strong containment measures (maximum observed CHI at 68.57) or temporarily for 8 weeks, doubling VCR, and combinations of high CHI and doubling VCR. All scenarios except the first one were simulated to take actions at week 4 and impact observed at week 6 due to 2-week lag.

We assessed the simulated impact of each scenario by comparing the predicted outcomes with observed hospitalizations. We used 1000 bootstrap simulations to generate 95% CIs. The results were reported as weekly, cumulative, and percentage reductions.

## Supporting information

S1 TextTable A in S1 Text.False Positive Rate (FPR, in %) at Different TPR Thresholds for Different Feature Sets. Fig A in S1 Text: Six sets of fitted chains. Set 1. B.1.1.7 (Alpha) variant. Period: 2020W51 to 2021W33. Set 2. B.1.617.2 (Delta) variant. Period: 2021W19 to 2022W06. Set 3. B.1.1.529 (Omicron) variant. Period: 2021W48 to 2022W17. Set 4. BA.2 (Omicron) variant. Period: 2021W50 to 2022W28. Set 5. BA.5 (Omicron) variant. Period: 2021W50 to 2022W28. Set 6. XBB (Omicron) variant. Period: 2022W52 to 2024W00. Fig B in S1 Text. Evolution of the a, b, and L parameters and their derivatives over increasing time windows (number of weeks considered). Top two rows refer to a dominant chain (France, chain 221), and bottom two to a non-dominant chain (Denmark, chain 169). Fig C in S1 Text. Receiver Operating Characteristic (ROC) curves for the deep learning model under different dataset restrictions and feature sets. Rows represent dataset scenarios: Case 1 (no restriction), Case 2 (pct_chain_n_1 < 0.17), and Case 3 (pct_chain_n_1 < 0.02). Columns compare three feature configurations: full feature set (including prevalence and sigmoid-based parameters), prevalence + a_3, b_3, L_3, and prevalence only. The model achieves excellent discrimination performance, especially when growth-related parameters are included. Fig D in S1 Text. Confusion matrices for the deep learning model across all dataset scenarios. Each matrix reports the classification performance under the three levels of data restriction (Cases 1–3) and the three feature sets. Including growth-related parameters leads to a reduction in false positives, especially in the most restrictive scenarios, highlighting the importance of early dynamics for robust prediction. Fig E in S1 Text. Real-world validation of DNN performance. Prediction score distributions for real-world predominant and transient chains, together with the corresponding confusion matrix. The separation of score distributions indicates the classifier’s ability to distinguish between the two classes on independent epidemiological data. Fig F in S1 Text. Fit procedure applied to key parameters distributions for predominant chains. The parameter a was fitted with a Gaussian distribution, the parameter b with a parabolic distribution, the parameter L with a Gaussian distribution and the parameter pct_chain_n_1 with a lognorm distribution Fig G in S1 Text. Distribution fitting excluding France (predominant chains). Parameter distributions and fitted curves obtained for predominant chains after excluding France from the dataset. The stability of fitted distribution parameters indicates robustness of the modeling procedure to country-level sampling variation. Fig H in S1 Text. Distribution fitting for transient chains dataset. Parameter distributions and fitted curves for transient chains. Wx denotes the prevalence percentage of a given chain at week x. The results illustrate the variability in early decay dynamics across transient viral chains. Fig I in S1 Text. Transient chain fitting excluding France. Parameter distributions for transient chains after excluding France from the dataset. Wx represents the prevalence percentage of the chain at week x. The fitting results remain consistent with the full dataset, indicating robustness of transient-chain parameter inference. Fig J in Supplementary text. Reinfection percentage among reported COVID-19 cases, containment and health index, and up-to-date COVID-19 vaccination coverage of Denmark. Fig K in S1 Text. Histogram showing the frequency distribution of the variable CH_week, which captures the interaction between containment and health index and the week counter of chains for Denmark from July 2020 to January 2024 (scaled 0–100). Fig L in S1 Text. Performance Evaluation of CatBoost model for predicting weekly hospitalizations in Denmark. Left panel: Actual vs. predicted values, showing a strong linear pattern indicating high prediction accuracy. Right panel: Residuals vs. predicted values, where the random spread around zero (red dashed line) suggests homoscedasticity, unbiased errors, and a proper model fit. Fig M in S1 Text. SHAP values illustrate variable impact on CatBoost model predictions for weekly hospitalizations in Denmark. Variables are ranked by contribution. High variable values with positive SHAP values indicate positive association, while those with negative SHAP values indicate negative association. Fig N in S1 Text. Partial dependence plots of selected variables showing the impact of each variable on CatBoost model predictions for weekly hospitalizations in Denmark. Fig O in S1 Text. Partial dependence plots of selected variables showing the impact of each variable on CatBoost model predictions for weekly hospitalizations in Denmark. Fig P in S1 Text. Observed hospitalizations and simulation results across various intervention scenarios for weekly and cumulative hospitalizations generated using CatBoost model, from chain 20 to chain 105 in Denmark. Fig Q in S1 Text. Observed hospitalizations and simulation results across various intervention scenarios for weekly and cumulative hospitalizations generated using CatBoost model, from chain 123 to chain 257 in Denmark. Fig R in S1 Text. Observed hospitalizations and simulation results across various intervention scenarios for weekly and cumulative hospitalizations generated using CatBoost model, from chain 265 to chain 418 in Denmark. Fig S in S1 Text: Performance of the Catboost models with different features for prediction of weekly hospitalizations due to variants in Denmark. Fig T in S1 Text: Important features of the Catboost models with different proportion of chains for prediction of weekly hospitalizations due to variants in Denmark. Fig U in S1 Text. Visual representation of the derived parameters used to characterize the growth dynamics of a SARS-CoV-2 variant based on a sigmoid curve. The logistic function models the weekly proportion of genomes attributed to a given variant. L represents the upper asymptote (maximum prevalence). The slope parameter a corresponds to the growth rate, while b indicates the inflection point of the curve. Temporal metrics include: $t_{\text{0}}$ (delay from first occurrence to algorithmic detection), $t_{\text{1}\text{0}\%}$ (time to reach 10% prevalence), $t_{react}$ (reaction time), and $t_{\text{1}\text{0}\%-\text{9}\text{0}\%}$ (time span between 10% and 90% prevalence). Early parameters a, b and L obtained at week 3 and at week 4 are also highlighted in the figure. Fig V in S1 Text. Percentage of prevalence at Week 1 vs Duration of real Transient chains. Real transient chains were classified based on their duration and initial prevalence. The prevalence vs duration distribution highlighted two different phase space regions. A cut on this distribution has been applied using an exponential threshold function defining two transient chain groups: above-threshold (showing some growth tendency) and below-threshold (typically vanishing early).(DOCX)
