# Supplementary material for: From sequences to strategies: Early detection of new SARS-CoV-2 variants via genetic distance to reduce hospitalizations
Source: PLoS Comput Biol. 2026 Sep 1;22(9):e1014707. doi: 10.1371/journal.pcbi.1014707 (PMC13577520; doi:10.1371/journal.pcbi.1014707)
Supplement: Supplementary file 1 [file pcbi.1014707.s001.docx]

# Supplementary materials

**Title**: From Sequences to Strategies: Early Detection of New SARS-CoV-2 Variants via Genetic Distance to Reduce Hospitalizations

**Short title:** Variant detection and impact of genetic distances

**Journal**: PLOS Computational Biology

**Authors**: Marika D’Avanzo^1,2,^*, Aung Pone Myint^3,^*, Giacomo Cacciapaglia^4,5^, Stefan Hohenegger^6^, Francesco Conventi^2,7^, Marta Nunes^3^

**Affiliations**

1. PhD National Programme in One Health approaches to infectious diseases and life science research, Department of Public Health, Experimental and Forensic Medicine, University of Pavia, Pavia, Italy.

2. INFN Sezione di Napoli, Complesso Universitario di Monte S. Angelo Edificio 6, Via Cintia, Naples, Italy.

3. Center of Excellence in Respiratory Pathogens (CERP), Hospices Civils de Lyon (HCL) and Centre International de Recherche en Infectiologie (CIRI), Équipe Santé Publique, Épidémiologie et Écologie Évolutive des Maladies Infectieuses (PHE3ID), Inserm U1111, CNRS UMR5308, ENS de Lyon, Université Claude Bernard Lyon 1, Lyon, France.

4. Laboratoire de Physique Théorique et Hautes Energies (LPTHE), UMR 7589, Sorbonne Université & CNRS, France.

5. Quantum Theory Center (ℏQTC) at IMADA & D-IAS, Southern Denmark Univ., Odense M, Denmark.

6. Université Claude Bernard Lyon 1, CNRS/IN2P3, IP2I UMR 5822, Villeurbanne, France.

7. Dipartimento di Ingegneria, Università degli studi di Napoli Parthenope, Centro Direzionale di Napoli, Naples, Italy.

* These authors contributed equally to this article and share first authorship.

**Correspondence**: Marta Nunes - [marta.nunes@chu-lyon.fr](mailto:marta.nunes@chu-lyon.fr)

**Table A.** False Positive Rate (FPR, in %) at Different TPR Thresholds for Different Feature Sets

| Case | Features | FPR @ 95% TPR (%) | FPR @ 99% TPR (%) | FPR @ 99.9% TPR (%) |
| --- | --- | --- | --- | --- |
| Case 1 | Full feature set | 0.00 | 0.00 | 0.00 |
| (no restriction) | Prevalence + $a_{3}$, $b_{3}$, $L_{3}$ | 0.00 | 0.00 | 5.93 |
|  | Prevalence only | 0.00 | 4.82 | 52.03 |
| Case 2 | Full feature set | 0.00 | 0.00 | 0.10 |
| ($pct\_chain\_n\_1<0.15$) | Prevalence + $a_{3}$, $b_{3}$, $L_{3}$ | 0.00 | 0.05 | 0.25 |
|  | Prevalence only | 1.46 | 4.07 | 45.20 |
| Case 3 | Full feature set | 0.00 | 0.00 | 0.10 |
| ($pct\_chain\_n\_1<0.02$) | Prevalence + $a_{3}$, $b_{3}$, $L_{3}$ | 0.05 | 0.05 | 0.15 |
|  | Prevalence only | 1.06 | 6.68 | 35.20 |

Supplementary Figures

**Fig A: Six sets of fitted chains.** Set 1. B.1.1.7 (Alpha) variant. Period: 2020W51 to 2021W33. Set 2. B.1.617.2 (Delta) variant. Period: 2021W19 to 2022W06. Set 3. B.1.1.529 (Omicron) variant. Period: 2021W48 to 2022W17. Set 4. BA.2 (Omicron) variant. Period: 2021W50 to 2022W28. Set 5. BA.5 (Omicron) variant. Period: 2021W50 to 2022W28. Set 6. XBB (Omicron) variant. Period: 2022W52 to 2024W00.

**Fig B. Evolution of the** $\boldsymbol{a}$**,** $\boldsymbol{b}$**, and** $\boldsymbol{L}$ **parameters and their derivatives over increasing time windows (number of weeks considered).** Top two rows refer to a dominant chain (France, chain 221), and bottom two to a non-dominant chain (Denmark, chain 169).

**Fig C. Receiver Operating Characteristic (ROC) curves for the deep learning model under different dataset restrictions and feature sets.** Rows represent dataset scenarios: Case 1 (no restriction), Case 2 (pct_chain_n_1<0.17), and Case 3 (pct_chain_n_1<0.02). Columns compare three feature configurations: full feature set (including prevalence and sigmoid-based parameters), prevalence + a_3, b_3, L_3, and prevalence only. The model achieves excellent discrimination performance, especially when growth-related parameters are included.

**Fig D. Confusion matrices for the deep learning model across all dataset scenarios.** Each matrix reports the classification performance under the three levels of data restriction (Cases 1–3) and the three feature sets. Including growth-related parameters leads to a reduction in false positives, especially in the most restrictive scenarios, highlighting the importance of early dynamics for robust prediction.

**Fig E. Real-world validation of DNN performance.** Prediction score distributions for real-world predominant and transient chains, together with the corresponding confusion matrix. The separation of score distributions indicates the classifier’s ability to distinguish between the two classes on independent epidemiological data.

**Fig F. Fit procedure applied to key parameters distributions for predominant chains.** The parameter a was fitted with a Gaussian distribution, the parameter b with a parabolic distribution, the parameter L with a Gaussian distribution and the parameter $pct\_chain\_n\_1$ with a lognorm distribution.

**Fig G. Distribution fitting excluding France (predominant chains).**
Parameter distributions and fitted curves obtained for predominant chains after excluding France from the dataset. The stability of fitted distribution parameters indicates robustness of the modeling procedure to country-level sampling variation**.**

**Fig H. Distribution fitting for transient chains dataset.** Parameter distributions and fitted curves for transient chains. Wx denotes the prevalence percentage of a given chain at week x. The results illustrate the variability in early decay dynamics across transient viral chains**.**

**Fig I. Transient chain fitting excluding France.** Parameter distributions for transient chains after excluding France from the dataset. Wx represents the prevalence percentage of the chain at week x. The fitting results remain consistent with the full dataset, indicating robustness of transient-chain parameter inference**.**

**Fig J. Reinfection percentage among reported COVID-19 cases, containment and health index, and up-to-date COVID-19 vaccination coverage of Denmark.**

**Fig K. Histogram showing the frequency distribution of the variable CH_week, which captures the interaction between containment and health index and the week counter of chains for Denmark from July 2020 to January 2024 (scaled 0 - 100).**

**Fig L. Performance Evaluation of CatBoost model for predicting weekly hospitalizations in Denmark.** Left panel: Actual vs. predicted values, showing a strong linear pattern indicating high prediction accuracy. Right panel: Residuals vs. predicted values, where the random spread around zero (red dashed line) suggests homoscedasticity, unbiased errors, and a proper model fit.

**Fig M. SHAP values illustrate variable impact on CatBoost model predictions for weekly hospitalizations in Denmark.** Variables are ranked by contribution. High variable values with positive SHAP values indicate positive association, while those with negative SHAP values indicate negative association.

**Fig N. Partial dependence plots of selected variables showing the impact of each variable on CatBoost model predictions for weekly hospitalizations in Denmark, while holding all other variables constant**.

**Fig O. Partial dependence plots of selected variables showing the impact of each variable on CatBoost model predictions for weekly hospitalizations in Denmark, while holding all other variables constant.**

**Fig P. Observed hospitalizations and simulation results across various intervention scenarios for weekly and cumulative hospitalizations generated using CatBoost model, from chain 20 to chain 105 in Denmark.**

**Fig Q. Observed hospitalizations and simulation results across various intervention scenarios for weekly and cumulative hospitalizations generated using CatBoost model, from chain 123 to chain 257 in Denmark.**

**Fig R. Observed hospitalizations and simulation results across various intervention scenarios for weekly and cumulative hospitalizations generated using CatBoost model, from chain 265 to chain 418 in Denmark.**

 **Fig S: Performance of the Catboost models with different features for prediction of weekly hospitalizations due to variants in Denmark. MAE - Mean Absolute Error, RMSE - Root Mean Squared Error, and MAPE - M** **ean Absolute Percentage Error.**

**Fig T: Important features of the Catboost models with different proportion of chains for prediction of weekly hospitalizations due to variants in Denmark. L0 – main model, L1 – one week-lagged proportions of chains, L2 – two week-lagged proportions of chains.**

**Fig U. Visual representation of the derived parameters used to characterize the growth dynamics of a SARS-CoV-2 variant based on a sigmoid curve.** The logistic function models the weekly proportion of genomes attributed to a given variant. $L$ represents the upper asymptote (maximum prevalence). The slope parameter $a$ corresponds to the growth rate, while $b$ indicates the inflection point of the curve. Temporal metrics include: $t_{0}$ (delay from first occurrence to algorithmic detection), $t_{10\%}$ (time to reach 10% prevalence), $t_{react}$ (reaction time), and $t_{10\%-90\%}$ (time span between 10% and 90% prevalence). Early parameters a, b and L obtained at week 3 and at week 4 are also highlighted in the figure.

**Fig V. Percentage of prevalence at Week 1 vs Duration of real Transient chains.** Real transient chains were classified based on their duration and initial prevalence. The prevalence vs duration distribution highlighted two different phase space regions. A cut on this distribution has been applied using an exponential threshold function defining two transient chain groups: above-threshold (showing some growth tendency) and below-threshold (typically vanishing early).
