# Supplementary material for: From sequences to strategies: Early detection of new SARS-CoV-2 variants via genetic distance to reduce hospitalizations
Source: PLoS Comput Biol. 2026 Sep 1;22(9):e1014707. doi: 10.1371/journal.pcbi.1014707 (PMC13577520; doi:10.1371/journal.pcbi.1014707)
Supplement: Supplementary file 2 [file pcbi.1014707.s002.docx]

**Peer Review Report**

**Manuscript Number:** PCOMPBIOL-D-26-00316

**Title:** From Sequences to Strategies: Early Detection of New SARS-CoV-2 Variants via Genetic Distance to Reduce Hospitalizations

**Journal:** PLOS Computational Biology

**Specific Comments**

**1. Reproducibility and external validation are insufficiently demonstrated** The pipeline relies on Levenshtein-distance–based clustering (“chains”) and a DNN trained on early growth features to predict whether a chain becomes dominant. However, the manuscript does not fully specify the chain construction/merging criteria. Furthermore, the DNN was trained using data augmentation (synthetic data), and its generalizability was not adequately proven on real-world out-of-sample historical data that were completely excluded from the distribution fitting process. It also does not convincingly quantify sensitivity to country-specific sequencing delays, sampling bias, and changing sequencing volume over time.

**Recommendation:** Please add: (i) clearer, implementation-level definitions for chain matching/aggregation; (ii) stronger out-of-sample validation (e.g., leave-one-country-out, or train on earlier periods and test on later periods, clearly distinguishing the performance on real versus synthetic data); and (iii) ablation/sensitivity analyses showing performance degradation under reduced sequencing coverage.

**2. Hospitalization attribution assumptions may introduce systematic bias and limit generalizability** For the CatBoost model based on Danish data, the approach assumes the weekly sequencing proportion of a chain directly reflects its proportion among hospitalized cases. However, this may not hold under differential testing, hospitalization delays, age structure, vaccination status, and non-representative sequencing of severe cases. In addition, fixing the Containment and Health Index (CHI) as a constant after 2022 may affect downstream scenario conclusions. Furthermore, the hospitalization model relies solely on Denmark, a country with an exceptionally high sequencing rate, which may not represent other nations.

**Recommendation:** Please provide sensitivity analyses using alternative attribution schemes and lag structures (and, if possible, stratified or proxy-adjusted analyses). Explicitly discuss in the text how these assumptions could introduce systematic bias and shift feature importance rankings (CatBoost) and the estimated hospitalization impacts. If data from other countries cannot be introduced for validation, please explicitly discuss in the Discussion section how differences in healthcare systems and reporting biases across countries might affect the model's cross-national generalizability.

**3. Limitations in Feature Importance and Causal Simulations** According to Figure 5, the most important feature in the CatBoost model is the "Week counter of the chain (week_num)", contributing 26.5%. This is essentially a proxy variable for the passage of time and is highly collinear with the accumulation of natural population immunity, the spread of vaccination, and changes in testing policies. Furthermore, the authors utilized CatBoost (a correlation-based machine learning regression model) to perform counterfactual simulations (e.g., doubling the vaccination coverage, maintaining sustained high CHI).

**Recommendation:** Machine learning models capture correlations, not direct causal relationships. Directly modifying a feature (like CHI or VCR) to simulate changes in hospitalization rates is highly risky in epidemiology. The authors need to explicitly acknowledge this methodological limitation in both the Methods and Discussion sections (i.e., that the model lacks rigorous causal inference modeling, such as Directed Acyclic Graphs [DAGs]) and explain whether "week_num" as the top feature masks true changes in the intrinsic pathogenicity of the virus itself.

**4. Figure Quality, Data Presentation, and Formatting Consistency** The readability of several figures is insufficient, and there are formatting inconsistencies in the main text, which do not meet the publication standards of *PLOS Computational Biology*.

**Recommendation:**

**Figure 1:** The axis labels, tick marks, and legend fonts in the subplots are too small to read easily. Please significantly enlarge the fonts and standardize the Y-axis scale across all subplots to facilitate cross-country comparison.

**Figure 5:** The variable abbreviations in the bar chart (e.g., pct_reif, HR_hosp) lack detailed epidemiological definitions. Please explicitly list the mathematical definitions and data sources for all CatBoost input features in the "Methods" section.

**Figure 6:** Given the very wide confidence intervals (CIs), it is recommended to explicitly state in the figure caption which scenarios have CIs that cross 0 (i.e., no statistically significant change) and to describe the absolute benefits of the simulation results more cautiously in the main text.

**Formatting:** Please conduct a comprehensive check of the subheading formats throughout the manuscript. For instance, "Cross-Country Variant Surveillance" on line 310 uses Title Case, whereas other subheadings at the same level within the Methods section (e.g., lines 302 and 328) use Sentence case. Ensure that typography (e.g., bolding, capitalization) is perfectly consistent.
