# Supplementary material for: From sequences to strategies: Early detection of new SARS-CoV-2 variants via genetic distance to reduce hospitalizations
Source: PLoS Comput Biol. 2026 Sep 1;22(9):e1014707. doi: 10.1371/journal.pcbi.1014707 (PMC13577520; doi:10.1371/journal.pcbi.1014707)
Supplement: Supplementary file 3 [file pcbi.1014707.s003.docx]

Dear Editorial office,

We are grateful for the opportunity to revise and resubmit our manuscript entitled “From Sequences to Strategies: Early Detection of New SARS-CoV-2 Variants via Genetic Distance to Reduce Hospitalizations”.

Please find appended the revised version of the manuscript, and below a point-by-point reply to the comments received.

We hope that this version is acceptable for publication, but we remain at your disposal if any further modifications are required.

Responses to reviewers questions

**Reviewer #1:**

**1. Reproducibility and external validation are insufficiently demonstrated** The pipeline relies on Levenshtein-distance–based clustering (“chains”) and a DNN trained on early growth features to predict whether a chain becomes dominant. However, the manuscript does not fully specify the chain construction/merging criteria. Furthermore, the DNN was trained using data augmentation (synthetic data), and its generalizability was not adequately proven on real-world out-of-sample historical data that were completely excluded from the distribution fitting process. It also does not convincingly quantify sensitivity to country-specific sequencing delays, sampling bias, and changing sequencing volume over time.

**Recommendation:** Please add: (i) clearer, implementation-level definitions for chain matching/aggregation; (ii) stronger out-of-sample validation (e.g., leave-one-country-out, or train on earlier periods and test on later periods, clearly distinguishing the performance on real versus synthetic data); and (iii) ablation/sensitivity analyses showing performance degradation under reduced sequencing coverage.

**Response**: We substantially expanded the Methods section to provide clearer implementation-level details on the chain construction and merging procedure. In particular, interrupted chains separated by 1–3 weeks were merged if they share the same dominant-sequence. A quality cut on the Ward-distance threshold (distance ≤ 100) has been applied consistently with the original framework described in page 4 of:

De Hoffer. F. Conventi ,*et al.* Variant-driven early warning via unsupervised machine learning analysis of spike protein mutations for COVID-19. *Sci Rep* **12**, 9275 (2022). <https://doi.org/10.1038/s41598-022-12442-8>)

To strengthen external validation, we added a new “Validation on real-world dataset” subsection in both the Results and Methods. The final DNN model was evaluated on 111 real chains (78 transient and 33 predominant). We also clarified throughout the manuscript the distinction between synthetic chains used for augmentation and real chains used for validation. The DNN results on real chains are in very good agreement with the one obtained with synthetic data and represent a strong validation of the data-augmentation procedure adopted in the paper.

For the leave-one-country-out validation we compared the DNN features statistical distribution obtained with the full dataset and the one obtained removing one country at time.

We found that the statistical distributions are compatible hence the fit procedure used to infer the feature pdf (probability density function) gives us very compatible results and the overall synthetic dataset used to train DNN are compatible with or without one single country confirming the DNN model stability. This has been checked both for predominant and transient chains.

Finally, we expanded the Discussion section to explicitly acknowledge limitations related to sequencing coverage fluctuations, sampling bias, and country-specific heterogeneity.

**2. Hospitalization attribution assumptions may introduce systematic bias and limit generalizability** For the CatBoost model based on Danish data, the approach assumes the weekly sequencing proportion of a chain directly reflects its proportion among hospitalized cases. However, this may not hold under differential testing, hospitalization delays, age structure, vaccination status, and non-representative sequencing of severe cases. In addition, fixing the Containment and Health Index (CHI) as a constant after 2022 may affect downstream scenario conclusions. Furthermore, the hospitalization model relies solely on Denmark, a country with an exceptionally high sequencing rate, which may not represent other nations.

**Recommendation:** Please provide sensitivity analyses using alternative attribution schemes and lag structures (and, if possible, stratified or proxy-adjusted analyses). Explicitly discuss in the text how these assumptions could introduce systematic bias and shift feature importance rankings (CatBoost) and the estimated hospitalization impacts. If data from other countries cannot be introduced for validation, please explicitly discuss in the Discussion section how differences in healthcare systems and reporting biases across countries might affect the model's cross-national generalizability.

**Response**: We added sensitivity analyses with delayed appearance of major variants for hospitalizations and the main important features remain the same.

The stratified or proxy-adjusted analyses could not be conducted due to data limitations.

CHI were monitored and reported until the end of 2022. It had been sustained at 21.43 from April to December 2022 as the containment measures applied during the pandemic were removed. The government policies after 2022 were not changed in limiting people movements or vaccination strategy, and we believed that the impact of the assumption of constant CHI will be negligible.

We made sure to include that the hospitalization analysis had limited generalizability due to the country' s uniqueness in the healthcare system.

**3. Limitations in Feature Importance and Causal Simulations** According to Figure 5, the most important feature in the CatBoost model is the "Week counter of the chain (week_num)", contributing 26.5%. This is essentially a proxy variable for the passage of time and is highly collinear with the accumulation of natural population immunity, the spread of vaccination, and changes in testing policies. Furthermore, the authors utilized CatBoost (a correlation-based machine learning regression model) to perform counterfactual simulations (e.g., doubling the vaccination coverage, maintaining sustained high CHI).

**Recommendation:** Machine learning models capture correlations, not direct causal relationships. Directly modifying a feature (like CHI or VCR) to simulate changes in hospitalization rates is highly risky in epidemiology. The authors need to explicitly acknowledge this methodological limitation in both the Methods and Discussion sections (i.e., that the model lacks rigorous causal inference modeling, such as Directed Acyclic Graphs [DAGs]) and explain whether "week_num" as the top feature masks true changes in the intrinsic pathogenicity of the virus itself.

**Response**: We added sensitivity analyses with different features in the models (time only and model without genetic distances) to show how the added values of those features.

The importance of week_num has been discussed.

**4. Figure Quality, Data Presentation, and Formatting Consistency** The readability of several figures is insufficient, and there are formatting inconsistencies in the main text, which do not meet the publication standards of *PLOS Computational Biology*.

**Recommendation:**

**Figure 1:** The axis labels, tick marks, and legend fonts in the subplots are too small to read easily. Please significantly enlarge the fonts and standardize the Y-axis scale across all subplots to facilitate cross-country comparison.

**Figure 5:** The variable abbreviations in the bar chart (e.g., pct_reif, HR_hosp) lack detailed epidemiological definitions. Please explicitly list the mathematical definitions and data sources for all CatBoost input features in the "Methods" section.

The mathematical definitions of the variables were added in the Methods section.

**Figure 6:** Given the very wide confidence intervals (CIs), it is recommended to explicitly state in the figure caption which scenarios have CIs that cross 0 (i.e., no statistically significant change) and to describe the absolute benefits of the simulation results more cautiously in the main text.

**Response**: We discussed that the simulations were purely theoretical scenarios.

**Formatting:** Please conduct a comprehensive check of the subheading formats throughout the manuscript. For instance, "Cross-Country Variant Surveillance" on line 310 uses Title Case, whereas other subheadings at the same level within the Methods section (e.g., lines 302 and 328) use Sentence case. Ensure that typography (e.g., bolding, capitalization) is perfectly consistent.

Edited.

**Reviewer #2:**

This manuscript presents an integrated framework for early detection of emerging SARS-CoV-2 variants based on spike-sequence clustering and temporal chain modeling, combined with downstream prediction of hospitalization burden using genetic distance, vaccination-related variables, and containment measures. The topic is timely and important, and the study addresses a relevant gap at the intersection of genomic surveillance and public health response. Overall, the manuscript is interesting and potentially impactful, but several methodological and interpretative issues should be addressed before publication.

General assessment

The main strengths of the manuscript are the attempt to move beyond retrospective lineage analysis toward early prediction of variant predominance, the extension of the clustering framework across multiple countries, and the effort to connect genetic distance measures with downstream hospitalization dynamics. The study is ambitious in scope and combines unsupervised clustering, deep learning classification, explainable boosting, and scenario simulations in a coherent narrative.
At the same time, the manuscript currently overstates some conclusions, especially regarding the relationship between genetic distance and hospitalization burden. While the predictive framework is promising, parts of the analysis rely on simplifying assumptions that limit causal interpretation and generalizability. In its current form, the paper would benefit from clearer methodological justification, stronger validation, and more cautious wording of the claims.

Major comments

1. Novelty is interesting, but the manuscript should better distinguish methodological innovation from integration of existing components.
The paper combines an existing chain-based clustering approach with a DNN for early classification and a CatBoost model for hospitalization prediction. This integrated design is valuable, but the manuscript should more clearly state what is truly novel here. From the current presentation, the main novelty seems to lie less in a fundamentally new algorithm and more in the combination of: early chain detection across six countries, prediction of chain predominance from early dynamics, and association of genetic distances with hospitalization outcomes.
This should be framed explicitly. At present, some passages may give the impression that the whole framework is entirely novel, while several components build directly on prior work.

**Response**: We revised the Introduction and Discussion to better clarify the novelty of the study. The manuscript now explicitly states that the main contribution lies in the integration of multiple components into a unified surveillance framework, namely: (i) extending chain-based surveillance across six countries, (ii) early prediction of predominant variants using a DNN trained on early dynamics, and (iii) associating genetic distance metrics with hospitalization-related epidemiological patterns.

2. The DNN evaluation strategy requires stronger validation

The DNN is trained on a dataset composed not only of real chains but also of a large number of synthetically generated predominant and transient chains. This is understandable given the scarcity of early-stage real examples, but it raises an important question: how well does performance on synthetic or semi-synthetic data translate to real-world prediction?

The manuscript reports very strong performance, even near-perfect classification in some settings, but this may partly reflect the fact that the augmented data follow the same generative assumptions used to define the problem. I strongly encourage the authors to provide a clearer validation focused on real chains only, even if sample size is limited. In particular:
How many real predominant and transient chains were used for training versus testing?
Was country-level separation explored, for example training on a subset of countries and testing on another?
Can the authors report performance on strictly real-world holdout examples without synthetic chains? Without such analyses, it is difficult to assess how robust the DNN would be in truly prospective surveillance settings.

**Response**: To better assess generalizability beyond synthetic augmentation, we added a dedicated “Validation on real-world dataset” subsection in the Results and Methods. The revised manuscript now clearly distinguishes between synthetic chains used during training and real-world chains used for validation. After excluding three chains with incomplete information (lack of week-3 information), the validation dataset consisted of 111 real chains. The final full-feature model achieved 97.3% accuracy and ROC-AUC = 0.980 on these real-world data, supporting the ability of the classifier to generalize to observed epidemiological dynamics. The DNN results on real chains are in very good agreement with the one obtained with synthetic data and represent a strong validation of the data-augmentation procedure adopted in the paper.

3. Potential data leakage or temporal optimism should be discussed more carefully
The paper aims to predict whether a chain will become predominant using features derived within the first 3–4 weeks. This is appropriate in principle, but the manuscript should clarify whether the feature construction, fitting procedure, and training setup fully preserve the temporal nature of the prediction task.

For example:
Are the sigmoid-derived parameters estimated using only information up to week 3 or week 4, or are they influenced by later observations?
Were synthetic chains generated independently of the final label-defining dynamics?
Are there any shared patterns across countries or waves that could make classification easier in hindsight than it would be prospectively?
A more explicit statement about how temporal leakage was avoided would strengthen the methodological credibility of the work.

**Response**: We clarified in the Methods section that all growth-related parameters used as DNN inputs were estimated exclusively using observations available within the first 3–4 weeks after chain detection. No information from later stages of chain evolution was used during feature construction. We also clarified that synthetic chains were generated independently from future observations beyond the prediction window. Moreover we checked that removing some of the available information (as for example removing all chains from a single country) the impact on the DNN features statistical distribution is negligible (Supplementary Fig S18, S19, S20).

4. The hospitalization analysis relies on strong assumptions that should be emphasized
The Danish hospitalization analysis is one of the most interesting parts of the manuscript, but also the most fragile from an inferential standpoint. The key assumption is that the proportion of sequences belonging to a given chain in a week reflects the proportion of hospitalizations attributable to that chain. This is a major simplification. Variant prevalence in the infected population does not necessarily map directly onto prevalence among hospitalized individuals, because hospitalization risk may differ by age, immunity status, prior infection history, comorbidity burden, testing patterns, and healthcare-seeking behavior.
The manuscript acknowledges this limitation, but in my view it should be emphasized more strongly and earlier, especially in the Results and Discussion. The current wording occasionally suggests a stronger link between genetic distance and hospitalization than the data can definitively support. The CatBoost model appears to capture useful predictive associations, but this should not be interpreted as demonstrating that genetic distance itself is a direct driver of hospitalization burden.

**Response**: We added sensitivity analyses with delayed appearance of major variants for hospitalizations and the main important features remain the same. The wordings were changed to correlation instead of causation.

5. The role of time is very strong and may confound the interpretation of genetic distance
In the CatBoost model, the detection week / week counter is the most important predictor, contributing more than any other variable. This suggests that a considerable portion of predictive performance may be driven by temporal structure, seasonality, and broad epidemiological context rather than by the genetic variables alone.
This is not a weakness per se, but it complicates the interpretation of LD1 and LD_vac. The manuscript should better disentangle whether these variables add substantial information beyond time. For example, the authors could report:
performance of a time-only baseline model,
performance gain when adding LD1 and LD_vac,
ablation analyses removing week-related variables,
and correlations among week, variant transitions, vaccination period, and genetic distance.
This would help readers understand whether genetic distance is independently informative or mainly proxying temporal regime shifts.

**Response**: We added sensitivity analyses with different features in the models (time only and model without genetic distances) to show how the added values of those features.

6. Scenario simulations are interesting but should be interpreted more cautiously
The counterfactual simulations are appealing from a public health perspective, particularly those involving better-matched vaccines or stronger CHI scenarios. However, these results should be framed clearly as model-based simulations, not as policy effect estimates.
Because the hospitalization model is observational and built on simplified assumptions, modifying inputs such as LD_vac, CHI, or VCR does not necessarily reflect what would happen under real interventions. In particular, changing vaccine genetic distance in silico is a highly abstract intervention that does not account for rollout timing, uptake heterogeneity, prior immunity, age structure, or waning immunity. The conclusions in the Abstract and Discussion should therefore be softened to avoid overinterpretation.

**Response**: We discussed that the simulations were purely theoretical scenarios.

7. Generalizability is limited
The surveillance analysis spans six countries, which is a strength. However, the hospitalization component is focused only on Denmark. This is understandable due to data availability, but the manuscript sometimes reads as if the conclusions about hospitalization burden are broadly generalizable. They are not yet.
The authors should clearly distinguish:
findings that appear robust across countries for chain detection and growth dynamics, and
findings that are demonstrated only in one country-specific hospitalization setting.
A stronger statement of this boundary would improve the manuscript.

**Response**: We made sure to include that the hospitalization analysis had limited generalizability due to the country' s uniqueness in the healthcare system.

8. Introducing more recent literature
The Introduction would benefit from a more comprehensive and updated positioning of the study within the recent literature. In the paragraph stating that “While COVID-19 vaccines have been shown to protect against infections and hospitalizations, the Omicron variant with extensive spike mutations has caused substantial hospitalizations despite lower intrinsic virulence”, the authors should consider adding further references to better support this contextual statement and to acknowledge more recent related work, including doi:10.1038/s41598-025-10132-9 and doi:10.1109/BIBM62325.2024.10822644.
In addition, when discussing previous studies in the Introduction, the literature review currently focuses on methods for early detection, anomaly detection, and lineage-frequency prediction. However, it should also acknowledge that recent studies have explored generative approaches aimed at producing plausible future viral sequences that may later become dominant, which is highly relevant to the broader landscape of prospective genomic surveillance. In this regard, the authors should cite doi:10.1093/bib/bbaf384. Including this line of work would provide a more complete overview of the field and better position the present study relative not only to detection/classification approaches, but also to sequence-generation frameworks for anticipating future dominant variants.

Minor comments
1. Clarify the definition of “predominant chain”
The manuscript defines predominant chains as those reaching at least 50% prevalence, but it would be useful to discuss why this threshold was selected and whether results are robust to alternative thresholds such as 30% or 40%.

**Response**: We clarified in the revised manuscript that the 50% prevalence threshold was selected to identify chains that became clearly predominant within the circulating viral population. Lower thresholds would include partially dominant or co-circulating variants, increasing ambiguity in the classification task.

2. Better explain the biological meaning of LD1 and LD_vac
These metrics are central to the paper, but a non-specialist reader may not immediately grasp why distance between consecutive chains and distance from the vaccine strain are expected to matter differently. A short conceptual explanation in the Introduction or Methods would help.

**Response**: We added an explanation about Levenshtein distance and what that distance means in the methods section.

3. More implementation details would improve reproducibility
The DNN architecture is described at a high level, but some details remain unclear, such as:
whether hyperparameters were tuned,
whether class imbalance handling was needed,
whether multiple random seeds were tested,
and how stable the results were across runs.
Similarly, more detail on CatBoost hyperparameter tuning and preprocessing would be welcome.

**Response**: We expanded the Methods section to provide additional implementation details regarding the DNN architecture, optimization procedure, training strategy, and CatBoost preprocessing. We also clarified that synthetic augmentation produced balanced datasets, therefore no additional class imbalance correction was required.

4. Figures are informative, but some could be made easier to interpret
Figure 1 and Figure 3 contain useful information, but are visually dense. Figure 3 in particular would benefit from a clearer explanation of the three cases and why the prevalence-only configuration performs worse. The manuscript text explains this, but the figure could be made more self-contained.

**Response**: We revised Figures 1 and 3 to improve readability by increasing font sizes and improving figure captions. In particular, Figure 3 now provides a clearer explanation of the different dataset configurations and feature combinations.

5. Language should be slightly moderated in some places
Expressions such as “we prove evidence” or wording implying direct public health actionability based on the current analyses should be softened. The study provides supporting evidence and a promising framework, but not definitive proof of intervention effects.

Suggested strengthening analyses
To improve the manuscript, I would strongly recommend the following additions if feasible:
A real-only validation of the DNN, even if limited in size.
An ablation study for the hospitalization model, especially comparing:
time-only,
time + vaccination/containment,
time + genetic distance,
full model.
Sensitivity analysis for the key assumptions linking sequence proportions to hospitalization proportions.
A clearer prospective framing of what information is available at the time of prediction.
More conservative discussion of the counterfactual simulations.

**Response**: A real-only validation of the DNN was added in the “Validation on real-world dataset” subsection in both the Results and Methods. The final DNN model was evaluated on 111 real chains (78 transient and 33 predominant). We added sensitivity analyses with different features in the models (time only and model without genetic distances) to show how the added values of those features. and the delayed appearance of variants in hospitalizations.

**Reviewer #3**:

his manuscript by D’Avanzo presents an integrated framework that combines sequence-based clustering, deep learning, and regression modeling to detect emerging SARS-CoV-2 variants and assess their impact on hospitalizations. The strongest aspect of the work is the authors’ attempts to combine clustering methods with a predictive model for variant dominance, followed by a separate model relating genetic features to hospitalizations. However, the methodology and the details of the models are not well described. I have trouble to understand what those models are, and reproducing the results are impossible. Additional details on model training and robustness are needed. I have several major concerns as I listed below.

One major concern is the strength of the claims regarding the performance of the deep learning model. The manuscript reports near-perfect classification of emerging variants within a very short time window, which is striking, but difficult to reconcile with the limited amount of real-world training data (in the order of hundreds, which is several orders of magnitude lower than total training data needed to train a DNN). A large portion of the training dataset appears to be generated through simulation, and it is not clear that these synthetic examples adequately capture the variability of real epidemiological dynamics. Without a clear demonstration that the model generalizes to unseen, real-world data, there is a substantial risk that the reported performance is overly optimistic.

**Response**: We agree that demonstrating generalization beyond synthetically generated chains is essential to support the robustness of the DNN framework. We addressed this concern through several modifications to both the manuscript text and the validation analyses.

First, we clarified that the original real-world dataset indeed contained a limited number of observed predominant and transient chains, motivating the use of synthetic augmentation to obtain sufficiently large datasets for DNN training.

We now explicitly distinguish throughout the manuscript between:

(i) synthetic chains used exclusively for data augmentation during training, and

(ii) real-world chains used for evaluation.

Second, to directly assess generalization to observed epidemiological dynamics, we added a new subsection entitled “Validation on real-world dataset” in the Results section, together with a corresponding methodological description in the Materials and Methods section. In this validation, the final classifier was evaluated on 111 real chains extracted from the six-country surveillance dataset (78 transient and 33 predominant chains), after excluding three chains with incomplete week-3 information that prevented reliable feature estimation.

The DNN model maintained very good performance on this real-world dataset and shown results on real chains that are in good agreement wrt synthetic dataset. Only three chains were misclassified overall. We additionally reported precision, recall, F1-score, confusion matrices, and prediction score distributions to provide a more complete characterization of model behavior on real epidemiological observations.

Third, we moderated several statements throughout the manuscript to avoid overstating the predictive performance of the DNN. In particular, expressions such as “near-perfect classification” were replaced with more cautious wording such as “high classification performance”.

Finally, we expanded the Discussion section to explicitly acknowledge the limitations of the synthetic augmentation strategy and the need for future prospective validation on fully independent country-level or temporally separated datasets.

A second major concern relates to the interpretation of the relationship between genetic distance and hospitalization. While the analysis identifies genetic distance metrics as important predictors, the model also shows that time (or week index) is the dominant feature. This raises the possibility that the observed associations are largely driven by broader temporal trends, such as seasonality, changes in population immunity, or evolving clinical practices, rather than genetic distance per se. At present, the manuscript does not sufficiently disentangle these effects. From a statistical point of view, the claims about genetic distance as a predictor of hospitalization risk should be presented more cautiously and framed as associations rather than causal relationships.

**Response**: We added sensitivity analyses with different features in the models (time only and model without genetic distances) to show how the added values of those features. And we edited the text using correlation instead of causation.

I am not sure if the assumption that sequence prevalence reflects the distribution of variants among hospitalized cases. In practice, sequencing data are subject to multiple biases, and variants can differ significantly in clinical severity. Using sequence proportions as a proxy for hospitalization burden may therefore introduce systematic errors/biases, particularly when comparing across variants or time periods. This limitation is acknowledged briefly, but its potential impact on the results is not investigated. More generally, the hospitalization analysis is restricted to Denmark, which limits the generalizability of the findings.

**Response**: We added sensitivity analyses with delayed appearance of major variants for hospitalizations and the main important features remain the same.

We made sure to include that the hospitalization analysis had limited generalizability due to the country' s uniqueness in the healthcare system.

The simulation results regarding reductions in hospitalization are simplistic and should be interpreted with caution. The reported reductions in hospitalizations under different intervention scenarios appear quite large, but they are derived from a single fitted model and depend on several assumptions. Given the uncertainties in both the input data and the modeling framework, these results are better viewed as illustrative rather than predictive.

**Response**: We discussed that the simulations were purely theoretical scenarios.

In terms of originality, the individual components of the approach are largely based on existing methods, and the main contribution lies in their combination.

**Response**: We revised the Introduction and Discussion to better distinguish between previously established methodological components and the novel contribution of the present work. In particular, we now explicitly state that the main novelty lies in the integration of: (i) cross-country chain-based surveillance, (ii) early prediction of predominant chains using deep learning based on early growth dynamics, and (iii) the association of genetic-distance metrics with hospitalization-related trends within a unified framework.

A couple of minor points include 1) I am not sure what “chains” exactly means 2) figure labels are too small for readers to see easily.

**Response**: We clarified the definition of “chains” in both the Introduction and Methods sections. Chains are now explicitly defined as a temporally connected series of clusters representing the longitudinal evolution of genetically related spike protein sequences across consecutive weeks. We also added additional implementation details regarding chain merging and continuity criteria.
