# Supplementary material for: From sequences to strategies: Early detection of new SARS-CoV-2 variants via genetic distance to reduce hospitalizations
Source: PLoS Comput Biol. 2026 Sep 1;22(9):e1014707. doi: 10.1371/journal.pcbi.1014707 (PMC13577520; doi:10.1371/journal.pcbi.1014707)
Supplement: Peer review history

# Peer review: Decision Letter 0

**DOI:** 10.1371/journal.pcbi.1014707.r001

**Authors:** Roger Dimitri Kouyos, Michael Domaratzki

20 Apr 2026

From Sequences to Strategies: Early Detection of New SARS-CoV-2 Variants via Genetic Distance to Reduce Hospitalizations

PLOS Computational Biology

Dear Dr. Nunes,

Thank you for submitting your manuscript to PLOS Computational Biology. After careful consideration, we feel that it has merit but does not fully meet PLOS Computational Biology's publication criteria as it currently stands. Therefore, we invite you to submit a revised version of the manuscript that addresses the points raised during the review process.

Please submit your revised manuscript by Jun 20 2026 11:59PM. If you will need more time than this to complete your revisions, please reply to this message or contact the journal office at ploscompbiol@plos.org. When you're ready to submit your revision, log on to https://www.editorialmanager.com/pcompbiol/ and select the 'Submissions Needing Revision' folder to locate your manuscript file.

Please include the following items when submitting your revised manuscript:

* A letter that responds to each point raised by the editor and reviewer(s). You should upload this letter as a separate file labeled 'Response to Reviewers'. This file does not need to include responses to formatting updates and technical items listed in the 'Journal Requirements' section below.

* A marked-up copy of your manuscript that highlights changes made to the original version. You should upload this as a separate file labeled 'Revised Manuscript with Track Changes'.

* An unmarked version of your revised paper without tracked changes. You should upload this as a separate file labeled 'Manuscript'.

If you would like to make changes to your financial disclosure, competing interests statement, or data availability statement, please make these updates within the submission form at the time of resubmission. Guidelines for resubmitting your figure files are available below the reviewer comments at the end of this letter

We look forward to receiving your revised manuscript.

Kind regards,

Michael Domaratzki

Academic Editor

PLOS Computational Biology

Roger Kouyos

Section Editor

PLOS Computational Biology

**Journal Requirements:**

1) Please ensure that the CRediT author contributions listed for every co-author are completed accurately and in full.

At this stage, the following Authors/Authors require contributions: Aung Pone Myint, Francesco Conventi, Giacomo Cacciapaglia, Marika D’Avanzo, Stefan Hohenegger, and Marta Nunes. Please ensure that the full contributions of each author are acknowledged in the "Add/Edit/Remove Authors" section of our submission form.

The list of CRediT author contributions may be found here: https://journals.plos.org/ploscompbiol/s/authorship#loc-author-contributions

2) Please upload all main figures as separate Figure files in .tif or .eps format. For more information about how to convert and format your figure files please see our guidelines:

https://journals.plos.org/ploscompbiol/s/figures

3) We have noticed that you have uploaded Supporting Information files, but you have not included a list of legends. Please add a full list of legends for your Supporting Information files after the references list.

4) Please amend your detailed Financial Disclosure statement. This is published with the article. It must therefore be completed in full sentences and contain the exact wording you wish to be published.

1) State what role the funders took in the study. If the funders had no role in your study, please state: "The funders had no role in study design, data collection and analysis, decision to publish, or preparation of the manuscript.".

Note: If the reviewer comments include a recommendation to cite specific previously published works, please review and evaluate these publications to determine whether they are relevant and should be cited. There is no requirement to cite these works unless the editor has indicated otherwise.

**Reviewers' comments:**

Reviewer's Responses to Questions

**Comments to the Authors:**

**Please note that one review is uploaded as an attachment.**

Reviewer #1: Please see the attached review for detailed comments.

Reviewer #2: This manuscript presents an integrated framework for early detection of emerging SARS-CoV-2 variants based on spike-sequence clustering and temporal chain modeling, combined with downstream prediction of hospitalization burden using genetic distance, vaccination-related variables, and containment measures. The topic is timely and important, and the study addresses a relevant gap at the intersection of genomic surveillance and public health response. Overall, the manuscript is interesting and potentially impactful, but several methodological and interpretative issues should be addressed before publication.

General assessment

The main strengths of the manuscript are the attempt to move beyond retrospective lineage analysis toward early prediction of variant predominance, the extension of the clustering framework across multiple countries, and the effort to connect genetic distance measures with downstream hospitalization dynamics. The study is ambitious in scope and combines unsupervised clustering, deep learning classification, explainable boosting, and scenario simulations in a coherent narrative.

At the same time, the manuscript currently overstates some conclusions, especially regarding the relationship between genetic distance and hospitalization burden. While the predictive framework is promising, parts of the analysis rely on simplifying assumptions that limit causal interpretation and generalizability. In its current form, the paper would benefit from clearer methodological justification, stronger validation, and more cautious wording of the claims.

Major comments

1. Novelty is interesting, but the manuscript should better distinguish methodological innovation from integration of existing components

The paper combines an existing chain-based clustering approach with a DNN for early classification and a CatBoost model for hospitalization prediction. This integrated design is valuable, but the manuscript should more clearly state what is truly novel here. From the current presentation, the main novelty seems to lie less in a fundamentally new algorithm and more in the combination of:

early chain detection across six countries, prediction of chain predominance from early dynamics,

and association of genetic distances with hospitalization outcomes.

This should be framed explicitly. At present, some passages may give the impression that the whole framework is entirely novel, while several components build directly on prior work.

2. The DNN evaluation strategy requires stronger validation

The DNN is trained on a dataset composed not only of real chains but also of a large number of synthetically generated predominant and transient chains. This is understandable given the scarcity of early-stage real examples, but it raises an important question: how well does performance on synthetic or semi-synthetic data translate to real-world prediction?

The manuscript reports very strong performance, even near-perfect classification in some settings, but this may partly reflect the fact that the augmented data follow the same generative assumptions used to define the problem. I strongly encourage the authors to provide a clearer validation focused on real chains only, even if sample size is limited. In particular:

How many real predominant and transient chains were used for training versus testing?

Was country-level separation explored, for example training on a subset of countries and testing on another?

Can the authors report performance on strictly real-world holdout examples without synthetic chains?

Without such analyses, it is difficult to assess how robust the DNN would be in truly prospective surveillance settings.

3. Potential data leakage or temporal optimism should be discussed more carefully

The paper aims to predict whether a chain will become predominant using features derived within the first 3–4 weeks. This is appropriate in principle, but the manuscript should clarify whether the feature construction, fitting procedure, and training setup fully preserve the temporal nature of the prediction task.

For example:

Are the sigmoid-derived parameters estimated using only information up to week 3 or week 4, or are they influenced by later observations?

Were synthetic chains generated independently of the final label-defining dynamics?

Are there any shared patterns across countries or waves that could make classification easier in hindsight than it would be prospectively?

A more explicit statement about how temporal leakage was avoided would strengthen the methodological credibility of the work.

4. The hospitalization analysis relies on strong assumptions that should be emphasized

The Danish hospitalization analysis is one of the most interesting parts of the manuscript, but also the most fragile from an inferential standpoint. The key assumption is that the proportion of sequences belonging to a given chain in a week reflects the proportion of hospitalizations attributable to that chain. This is a major simplification. Variant prevalence in the infected population does not necessarily map directly onto prevalence among hospitalized individuals, because hospitalization risk may differ by age, immunity status, prior infection history, comorbidity burden, testing patterns, and healthcare-seeking behavior.

The manuscript acknowledges this limitation, but in my view it should be emphasized more strongly and earlier, especially in the Results and Discussion. The current wording occasionally suggests a stronger link between genetic distance and hospitalization than the data can definitively support. The CatBoost model appears to capture useful predictive associations, but this should not be interpreted as demonstrating that genetic distance itself is a direct driver of hospitalization burden.

5. The role of time is very strong and may confound the interpretation of genetic distance

In the CatBoost model, the detection week / week counter is the most important predictor, contributing more than any other variable. This suggests that a considerable portion of predictive performance may be driven by temporal structure, seasonality, and broad epidemiological context rather than by the genetic variables alone.

This is not a weakness per se, but it complicates the interpretation of LD1 and LD_vac. The manuscript should better disentangle whether these variables add substantial information beyond time. For example, the authors could report:

performance of a time-only baseline model,

performance gain when adding LD1 and LD_vac,

ablation analyses removing week-related variables,

and correlations among week, variant transitions, vaccination period, and genetic distance.

This would help readers understand whether genetic distance is independently informative or mainly proxying temporal regime shifts.

6. Scenario simulations are interesting but should be interpreted more cautiously

The counterfactual simulations are appealing from a public health perspective, particularly those involving better-matched vaccines or stronger CHI scenarios. However, these results should be framed clearly as model-based simulations, not as policy effect estimates.

Because the hospitalization model is observational and built on simplified assumptions, modifying inputs such as LD_vac, CHI, or VCR does not necessarily reflect what would happen under real interventions. In particular, changing vaccine genetic distance in silico is a highly abstract intervention that does not account for rollout timing, uptake heterogeneity, prior immunity, age structure, or waning immunity. The conclusions in the Abstract and Discussion should therefore be softened to avoid overinterpretation.

7. Generalizability is limited

The surveillance analysis spans six countries, which is a strength. However, the hospitalization component is focused only on Denmark. This is understandable due to data availability, but the manuscript sometimes reads as if the conclusions about hospitalization burden are broadly generalizable. They are not yet.

The authors should clearly distinguish:

findings that appear robust across countries for chain detection and growth dynamics, and

findings that are demonstrated only in one country-specific hospitalization setting.

A stronger statement of this boundary would improve the manuscript.

8. Introducing more recent literature

The Introduction would benefit from a more comprehensive and updated positioning of the study within the recent literature. In the paragraph stating that “While COVID-19 vaccines have been shown to protect against infections and hospitalizations, the Omicron variant with extensive spike mutations has caused substantial hospitalizations despite lower intrinsic virulence”, the authors should consider adding further references to better support this contextual statement and to acknowledge more recent related work, including doi:10.1038/s41598-025-10132-9 and doi:10.1109/BIBM62325.2024.10822644.

In addition, when discussing previous studies in the Introduction, the literature review currently focuses on methods for early detection, anomaly detection, and lineage-frequency prediction. However, it should also acknowledge that recent studies have explored generative approaches aimed at producing plausible future viral sequences that may later become dominant, which is highly relevant to the broader landscape of prospective genomic surveillance. In this regard, the authors should cite doi:10.1093/bib/bbaf384. Including this line of work would provide a more complete overview of the field and better position the present study relative not only to detection/classification approaches, but also to sequence-generation frameworks for anticipating future dominant variants.

Minor comments

1. Clarify the definition of “predominant chain”

The manuscript defines predominant chains as those reaching at least 50% prevalence, but it would be useful to discuss why this threshold was selected and whether results are robust to alternative thresholds such as 30% or 40%.

2. Better explain the biological meaning of LD1 and LD_vac

These metrics are central to the paper, but a non-specialist reader may not immediately grasp why distance between consecutive chains and distance from the vaccine strain are expected to matter differently. A short conceptual explanation in the Introduction or Methods would help.

3. More implementation details would improve reproducibility

The DNN architecture is described at a high level, but some details remain unclear, such as:

whether hyperparameters were tuned,

whether class imbalance handling was needed,

whether multiple random seeds were tested,

and how stable the results were across runs.

Similarly, more detail on CatBoost hyperparameter tuning and preprocessing would be welcome.

4. Figures are informative, but some could be made easier to interpret

Figure 1 and Figure 3 contain useful information, but are visually dense. Figure 3 in particular would benefit from a clearer explanation of the three cases and why the prevalence-only configuration performs worse. The manuscript text explains this, but the figure could be made more self-contained.

5. Language should be slightly moderated in some places

Expressions such as “we prove evidence” or wording implying direct public health actionability based on the current analyses should be softened. The study provides supporting evidence and a promising framework, but not definitive proof of intervention effects.

Suggested strengthening analyses

To improve the manuscript, I would strongly recommend the following additions if feasible:

A real-only validation of the DNN, even if limited in size.

An ablation study for the hospitalization model, especially comparing:

time-only,

time + vaccination/containment,

time + genetic distance,

full model.

Sensitivity analysis for the key assumptions linking sequence proportions to hospitalization proportions.

A clearer prospective framing of what information is available at the time of prediction.

More conservative discussion of the counterfactual simulations.

Reviewer #3: This manuscript by D’Avanzo presents an integrated framework that combines sequence-based clustering, deep learning, and regression modeling to detect emerging SARS-CoV-2 variants and assess their impact on hospitalizations. The strongest aspect of the work is the authors’ attempts to combine clustering methods with a predictive model for variant dominance, followed by a separate model relating genetic features to hospitalizations. However, the methodology and the details of the models are not well described. I have trouble to understand what those models are, and reproducing the results are impossible. Additional details on model training and robustness are needed. I have several major concerns as I listed below.

One major concern is the strength of the claims regarding the performance of the deep learning model. The manuscript reports near-perfect classification of emerging variants within a very short time window, which is striking, but difficult to reconcile with the limited amount of real-world training data (in the order of hundreds, which is several orders of magnitude lower than total training data needed to train a DNN). A large portion of the training dataset appears to be generated through simulation, and it is not clear that these synthetic examples adequately capture the variability of real epidemiological dynamics. Without a clear demonstration that the model generalizes to unseen, real-world data, there is a substantial risk that the reported performance is overly optimistic.

A second major concern relates to the interpretation of the relationship between genetic distance and hospitalization. While the analysis identifies genetic distance metrics as important predictors, the model also shows that time (or week index) is the dominant feature. This raises the possibility that the observed associations are largely driven by broader temporal trends, such as seasonality, changes in population immunity, or evolving clinical practices, rather than genetic distance per se. At present, the manuscript does not sufficiently disentangle these effects. From a statistical point of view, the claims about genetic distance as a predictor of hospitalization risk should be presented more cautiously and framed as associations rather than causal relationships.

I am not sure if the assumption that sequence prevalence reflects the distribution of variants among hospitalized cases. In practice, sequencing data are subject to multiple biases, and variants can differ significantly in clinical severity. Using sequence proportions as a proxy for hospitalization burden may therefore introduce systematic errors/biases, particularly when comparing across variants or time periods. This limitation is acknowledged briefly, but its potential impact on the results is not investigated. More generally, the hospitalization analysis is restricted to Denmark, which limits the generalizability of the findings.

The simulation results regarding reductions in hospitalization are simplistic and should be interpreted with caution. The reported reductions in hospitalizations under different intervention scenarios appear quite large, but they are derived from a single fitted model and depend on several assumptions. Given the uncertainties in both the input data and the modeling framework, these results are better viewed as illustrative rather than predictive.

In terms of originality, the individual components of the approach are largely based on existing methods, and the main contribution lies in their combination.

A couple of minor points include 1) I am not sure what “chains” exactly means 2) figure labels are too small for readers to see easily.

**********

**Have the authors made all data and (if applicable) computational code underlying the findings in their manuscript fully available?**

The PLOS Data policy requires authors to make all data and code underlying the findings described in their manuscript fully available without restriction, with rare exception (please refer to the Data Availability Statement in the manuscript PDF file). The data and code should be provided as part of the manuscript or its supporting information, or deposited to a public repository. For example, in addition to summary statistics, the data points behind means, medians and variance measures should be available. If there are restrictions on publicly sharing data or code —e.g. participant privacy or use of data from a third party—those must be specified.

Reviewer #1: **No:**  The Data Availability Statement should be clarified and made fully consistent with PLOS data and code sharing requirements, particularly regarding code availability and any access restrictions associated with GISAID data.

Reviewer #2: Yes

Reviewer #3: None

**********

PLOS authors have the option to publish the peer review history of their article (what does this mean?). If published, this will include your full peer review and any attached files.

If you choose “no”, your identity will remain anonymous but your review may still be made public.

**Do you want your identity to be public for this peer review?**  For information about this choice, including consent withdrawal, please see our Privacy Policy.

Reviewer #1: No

Reviewer #2: **Yes:**  Rancati Simone

Reviewer #3: No

[NOTE: If reviewer comments were submitted as an attachment file, they will be attached to this email and accessible via the submission site. Please log into your account, locate the manuscript record, and check for the action link "View Attachments". If this link does not appear, there are no attachment files.]

**Figure resubmission:**

After uploading your figures to PLOS’s NAAS tool - https://ngplosjournals.pagemajik.ai/artanalysis, NAAS will process the files provided and display the results in the "Uploaded Files" section of the page as the processing is complete. If the uploaded figures meet our requirements (or NAAS is able to fix the files to meet our requirements), the figure will be marked as "fixed" above. If NAAS is unable to fix the files, a red "failed" label will appear above. When NAAS has confirmed that the figure files meet our requirements, please download the file via the download option, and include these NAAS processed figure files when submitting your revised manuscript.

**Reproducibility:**

To enhance the reproducibility of your results, we recommend that authors of applicable studies deposit laboratory protocols in protocols.io, where a protocol can be assigned its own identifier (DOI) such that it can be cited independently in the future. Additionally, PLOS ONE offers an option to publish peer-reviewed clinical study protocols. Read more information on sharing protocols at https://plos.org/protocols?utm_medium=editorial-email&utm_source=authorletters&utm_campaign=protocols

Attachment Submitted filename: Peer Review Report .docx

# Author comment: Author response to Decision Letter 1

**DOI:** 10.1371/journal.pcbi.1014707.r002

22 Jun 2026

Attachment Submitted filename: Response_to_Reviewers_19jun2026.docx

# Peer review: Decision Letter 1

**DOI:** 10.1371/journal.pcbi.1014707.r003

**Authors:** Roger Dimitri Kouyos, Michael Domaratzki

11 Aug 2026

Dear Dr. Nunes,

We are pleased to inform you that your manuscript 'From Sequences to Strategies: Early Detection of New SARS-CoV-2 Variants via Genetic Distance to Reduce Hospitalizations' has been provisionally accepted for publication in PLOS Computational Biology.

Before your manuscript can be formally accepted you will need to complete some formatting changes, which you will receive in a follow up email. A member of our team will be in touch with a set of requests.

Please note that your manuscript will not be scheduled for publication until you have made the required changes, so a swift response is appreciated.

IMPORTANT: The editorial review process is now complete. PLOS will only permit corrections to spelling, formatting or significant scientific errors from this point onwards. Requests for major changes, or any which affect the scientific understanding of your work, will cause delays to the publication date of your manuscript.

Should you, your institution's press office or the journal office choose to press release your paper, you will automatically be opted out of early publication. We ask that you notify us now if you or your institution is planning to press release the article. All press must be co-ordinated with PLOS.

Thank you again for supporting Open Access publishing; we are looking forward to publishing your work in PLOS Computational Biology.

Best regards,

Michael Domaratzki

Academic Editor

PLOS Computational Biology

Roger Kouyos

Section Editor

PLOS Computational Biology

***********************************************************

Dear Authors - thank you for your attention to the reviewers comments. You will see that two of the reviewers have indicated that the paper should be accepted. Unfortunately, Reviewer #1 (previously suggested "Minor Revision") has been unable to provide a review for the paper. However, I have gone through the details of the review and assured myself that the comments have been addressed.

Reviewer's Responses to Questions

**Comments to the Authors:**

**Please note here if the review is uploaded as an attachment.**

Reviewer #2: Thanks to address all my comments. The paper is now ready for the publication

Reviewer #3: I do not have further comments.

**********

**Have the authors made all data and (if applicable) computational code underlying the findings in their manuscript fully available?**

The PLOS Data policy requires authors to make all data and code underlying the findings described in their manuscript fully available without restriction, with rare exception (please refer to the Data Availability Statement in the manuscript PDF file). The data and code should be provided as part of the manuscript or its supporting information, or deposited to a public repository. For example, in addition to summary statistics, the data points behind means, medians and variance measures should be available. If there are restrictions on publicly sharing data or code —e.g. participant privacy or use of data from a third party—those must be specified.

Reviewer #2: Yes

Reviewer #3: None

**********

PLOS authors have the option to publish the peer review history of their article (what does this mean?). If published, this will include your full peer review and any attached files.

If you choose “no”, your identity will remain anonymous but your review may still be made public.

**Do you want your identity to be public for this peer review?**  For information about this choice, including consent withdrawal, please see our Privacy Policy.

Reviewer #2: **Yes:**  Rancati Simone

Reviewer #3: No

# Editor report: Acceptance letter

**DOI:** 10.1371/journal.pcbi.1014707.r004

**Authors:** Roger Dimitri Kouyos, Michael Domaratzki

PCOMPBIOL-D-26-00316R1

From Sequences to Strategies: Early Detection of New SARS-CoV-2 Variants via Genetic Distance to Reduce Hospitalizations

Dear Dr Nunes,

I am pleased to inform you that your manuscript has been formally accepted for publication in PLOS Computational Biology. Your manuscript is now with our production department and you will be notified of the publication date in due course.

The corresponding author will soon be receiving a typeset proof for review, to ensure errors have not been introduced during production. Please review the PDF proof of your manuscript carefully, as this is the last chance to correct any errors. Please note that major changes, or those which affect the scientific understanding of the work, will likely cause delays to the publication date of your manuscript.

Soon after your final files are uploaded, unless you have opted out, the early version of your manuscript will be published online. The date of the early version will be your article's publication date. The final article will be published to the same URL, and all versions of the paper will be accessible to readers.

For Research, Software, and Methods articles, you will receive an invoice from PLOS for your publication fee after your manuscript has reached the completed accept phase. If you receive an email requesting payment before acceptance or for any other service, this may be a phishing scheme. Learn how to identify phishing emails and protect your accounts at https://explore.plos.org/phishing.

Thank you again for supporting PLOS Computational Biology and open-access publishing. We are looking forward to publishing your work!

With kind regards,

Sharmila Kamatchi

PLOS Computational Biology | Carlyle House, Carlyle Road, Cambridge CB4 3DN | United Kingdom ploscompbiol@plos.org | Phone +44 (0) 1223-442824 | ploscompbiol.org | @PLOSCompBiol
